# Pathophysiology and Current Drug Treatments for Post-Stroke Depression: A Review

**DOI:** 10.3390/ijms232315114

**Published:** 2022-12-01

**Authors:** Dmitry Frank, Benjamin F. Gruenbaum, Alexander Zlotnik, Michael Semyonov, Amit Frenkel, Matthew Boyko

**Affiliations:** 1 Department of Anesthesiology and Critical Care, Soroka University Medical Center, Ben-Gurion of the Negev, Beer-Sheva 84105, Israel; 2 Department of Anesthesiology and Perioperative Medicine, Mayo Clinic, Jacksonville, FL 32224, USA

**Keywords:** drug therapies, glutamate, monoamines, pathogenesis, post-stroke depression (PSD)

## Abstract

Post-stroke depression (PSD) is a biopsychosocial disorder that affects individuals who have suffered a stroke at any point. PSD has a 20 to 60 percent reported prevalence among stroke survivors. Its effects are usually adverse, can lead to disability, and may increase mortality if not managed or treated early. PSD is linked to several other medical conditions, including anxiety, hyper-locomotor activity, and poor functional recovery. Despite significant awareness of its adverse impacts, understanding the pathogenesis of PSD has proved challenging. The exact pathophysiology of PSD is unknown, yet its complexity has been definitively shown, involving mechanisms such as dysfunction of monoamine, the glutamatergic systems, the gut-brain axis, and neuroinflammation. The current effectiveness of PSD treatment is about 30–40 percent of all cases. In this review, we examined different pathophysiological mechanisms and current pharmacological and non-pharmacological approaches for the treatment of PSD.

## 1. Introduction

Strokes occur due to interference in the supply of blood to the brain and are a major cause of global morbidity and mortality. Research from the American Heart Association shows that there are about 700,000 strokes annually in the United States alone [1]. Failure to identify and rectify this condition can lead to irreversible tissue damage.

Post-stroke depression (PSD) is the most frequent neuropsychiatric comorbidity of stroke, capable of affecting more than a third of stroke survivors [2]. The PSD prevalence rate currently stands between 20 and 60 percent. Additional findings show that depression is more common among stroke patients than those suffering from physical impairments resulting from orthopedic injuries [2]. The Canadian Stroke Best Practices research shows that all individuals who have experienced a stroke are at a high risk of post-stroke depression [3]. Although PSD is known to have a detrimental effect on functional recovery and quality of life after stroke, it is often overlooked and untreated.

Patients diagnosed with PSD always present with depressed moods coupled with a loss of interest or pleasure in activities. Given that this depression immediately follows a stroke, affected people also exhibit several depressive symptoms, including psychomotor agitation, feelings of worthlessness, sleep disturbances, weight fluctuations, and suicidal ideation, which can last for more than two weeks [1]. Most patients begin showing signs of depression three months following a stroke, though symptoms of the disorder can be exhibited by the affected individuals at any stage of recovery.

The exact pathophysiology of PSD is unknown, yet its complexity has been definitively shown, involving mechanisms such as dysfunction of monoamine, the glutamatergic systems, and the gut-brain axis, HLA and related to neuroinflammation, reducing BDNF expressing as well. [4]. According to the monoamine theory, ischemic lesions in the brain cause unprecedented interruptions of the biogenic amine-containing axons, which ascend from the brainstem nuclei as it extends to the cerebral cortex [5]. Dysfunctions in glutamatergic neurotransmission can also cause the occurrence of many psychiatric conditions and abnormalities, including major depressive disorder [4]. On the other hand, neuroinflammation involves astrocytes, microglia (sentinel cells of innate immunity in the brain), cytokines (tumor necrosis factor-alpha interleukin 1, interleukin 6), microRNAs, and monocyte-derived macrophages to the pathogenesis [6]. Tubb et al. [7] show that dysregulation of immune function is linked to infectious diseases and psychiatric disorders such as depression. Li et al. [8] also supported the correlation between DNA methylation, BDNF, and depression, ascertaining that BDNF and nuclear receptor subfamily 3 group C member 1 (NR3C1) gene methylation levels are linked to depressive symptoms.

Despite the availability of information on the harmful effects of this disorder on the recovery of patients after stroke, little progress has been achieved in developing a universal and reliable treatment for PSD [9]. Conroy et al. [10] argue that depression among patients with neurological disorders is often more challenging to treat than depression suffered by the general population. A recent study established that suicide rates among patients who have had a stroke are increased compared with the general population [10]. Thus, it is essential for stroke survivors and their family members to be provided with the necessary education and information on the impact of the condition and better ways to manage it at all stages of care.

PSD exposes individuals to suboptimal recovery risks, poor quality of life, poor functional outcomes, and mortality in adverse cases [11]. Screening for post-stroke depression is usually conducted in medically appropriate cases while considering the cognitive diagnoses of those stroke survivors. It is advisable for trained medical professionals undertaking the process to exclude unresponsive patients. Additionally, individuals with inherent mental or physical health deficits capable of interfering with the screening for mood disorders should also be excluded from the screening. The screening should include an evaluation of risk factors of PSD, be implemented at various stages of stroke care, and be designed individually for each patient. Despite the evidence that PSD is one of the most severe complications after a stroke, few medical guidelines are available on preventing or treating the condition. This review addresses new drug treatments for PSD while summarizing the latest scholarly and scientifically published evidence on the best screening, management, and treatment methods.

## 2. Association between PSD and Post-Stroke Anxiety

Depression and anxiety are linked as among the common conditions that are likely to arise after an individual suffers a stroke. Even though it is normal for the affected individuals to feel sad, anxious, and worried following a stroke, the feelings are supposed to be transient and go away when the stressful situation is over. Findings from the Stroke Foundation point out that the persistence of PSD and post-stroke anxiety (PSA) symptoms for more than two weeks causes depression and anxiety [12]. The signs and feelings are usually overwhelming and challenging to control, thus making it hard for a person to cope daily.

Schottke and Giabbiconi [12] found a significant correlation between PSD prevalence and PSA prevalence. The two disorders are primarily associated with the experiences and consequences of the stroke itself. Another study by Fang et al. [13] showed that about 30 percent of stroke survivors had depressive symptoms while 20–25 percent experience signs of anxiety. Though PSD and PSA share a vital link, the predictors and diagnosis of the conditions vary slightly. PSA is less related to stroke than PSD since its primary predictors comprise lifetime anxiety disorders, including obsessive-compulsive disorder (OCD) and agoraphobia without panic disorder. In contrast, PSD cannot be predicted by lifetime depression. Therefore, PSD is less predictable than PSA as a post-stroke affective disorder because it is stroke-related [12]. However, both PSD and PSA may result in adverse neurological and functional deficits if psychological interventions are not implemented in time. The relationship between PSD and PSA can be best elaborated by looking into the rehabilitation services provided to patients suffering from the disorders, as well as shared diagnostics, comorbidity, severity, etiology, and risk factors.

### 2.1. Rehabilitation Services for PSD and PSA

The negative impacts resulting from PSD and PSA necessitate the need for rehabilitation services to offer help to those affected. However, PSD leads to poor functional and cognitive recovery, which at times makes it difficult for the affected person to participate in rehabilitation. PSA causes individuals to experience poor self-control, immobility, and fatigue [13]. Post-stroke rehabilitation is usually conducted to help stroke survivors to achieve the best overall outcome for their conditions. Unfortunately, it cannot offer the support needed to reverse any brain damage caused. However, individuals have the opportunity to relearn the skills they had lost when part of their brain was significantly damaged [14]. Neuro-rehabilitation programs for both PSD and PSA are carefully directed and well-focused. Due to the repetitive aspects of rehabilitation, it is individually customized to solve the specific needs of the affected persons.

Some skills impaired by a stroke that people can regain by adhering to the regimen proposed in their programs include an increase in sensation, ability to walk, improved coordination, and improved visual acuity [14]. Additionally, one also has the opportunity to learn new techniques of survival while living with any remaining disabilities, for example, through the use of assistive communication devices if language has been affected.

Paolucci et al. [15] argue that when not treated or managed quickly, PSD and PSA have the potential to increase disability, which is usually caused by neurological impairment, by more than fifteen percent in stroke survivors. To address patients’ psychological needs, psychological care is often among the services offered in rehabilitation centers. Most patients experience high levels of social stress, which can give rise to depression and anxiety. Examples of psychological stress include changes such as lower self-esteem and body image. These stressors make it difficult for patients to adjust without rehabilitative help or intervention [16,17]. Through rehabilitation, psychoeducation, and support from care providers, those affected with PSD and PSA are in a position to reduce or manage their physical pain effectively.

### 2.2. Diagnostics for PSD and PSA

Both PSD and PSD are diagnosed and defined by the Diagnostic and Statistical Manual of Mental Disorders Fifth Edition (DSM-5). The manual categorizes PSD and PSA disorders as including mixed mood features, major depressive-like episodes, and increasingly diminished individual interest in all-day activities [1,16,17]. However, there are some exceptional cases when DSM-5 is not used when diagnosing patients, especially for those who cannot describe their psychological conditions coherently and do not exhibit typical psychological symptoms. A stroke may cause speech and mental disabilities, adversely affecting communication ability. Thus, such people can only experience or convey physical symptoms such as insomnia, fatigue, and appetite loss [18].

It is often challenging for medical practitioners to classify such symptoms as mental problems since anyone can experience them after a stroke. As a result, additional measures and scales are used to bring optimal results. Other diagnosis methods include the Hamilton Depression Rating Scale (HDRS), the Patient Health Questionnaire (PHQ)-9, and the Center of Epidemiological Studies-Depression Scale (CESD) [19]. However, clinicians and medical professionals must apply sound judgment and check for issues such as sleep disturbances when using CESD, HDRS, and PHQ-9 since they are not diagnostic tools. These screening measures should only be used in conducting assessments on the psychological status of patients and then making referrals of suspected cases to specialists dealing with psychological issues for treatment [19]. A stroke is life-changing for any individual, given the difficulties of managing day-to-day activities effectively. However, a patient is considered to suffer from PSD and PSA if they experience a mood disorder with a significant depression-like or anxiety-like episode with symptoms lasting two or more weeks.

### 2.3. Risk Factors

Both PSD and PSA are prevalent after stroke and occur in about one-third of stroke survivors. However, despite their adverse effects, few studies have been published to explain the risks associated with the disorders. Possible risk factors for PSD that have been examined include genetic factors, demographic factors, fatigue, cognitive impairment, functional and cognitive impairment, and medical and psychiatric history [1,20]. The evidence, therefore, suggests that PSD and PSA are multifactorial and can affect patients’ activities and daily life [20].

Moreover, evidence strongly shows that stroke characteristics and lesion location can contribute to mental conditions. Robinson et al. [1] established that patients with left frontal and basal ganglia lesions have higher chances of developing PSD than those with other lesion locations. The appearance of PSD symptoms among people within left frontal and basal ganglia lesions locations is transient and restricted to the first two months after stroke [1]. These lesion locations are among the common and significant risk factors that clinicians can use for any association with PSD.

In addition, lesion-location analysis conducted by Li et al. [20] showed that patients with acute frontal lobe infarction had increased chances of having PSA. However, unlike PSD, it is impossible to locate a single location in the brain that is directly related to PSA. Several regions in the cerebral hemisphere have been implicated in the development of anxiety, including the corona radiate, internal capsule, and corpus callosum. The cerebral hemispheres usually contain neural circuits that modulate emotional regulation [20]. Thus, when the cerebral hemispheric white matter is affected by stroke, patients are more likely to suffer from PSA in the acute stages of stroke or three months after stroke. Depression is also a significant risk factor for PSA and may increase the severity of the symptoms of the disorder. Since most PSD and PSA risk factors are similar, medical professionals should critically assess them to mitigate their adverse effects.

## 3. Association between PSD and Post-Stroke Motor Activity

Motor activity is one of the skills that PSD-affected individuals cannot perform on their own without assistance from medical professionals. Besides suffering from psychological challenges, patients also experience physical symptoms such as fatigue and energy loss, making it difficult for them to move easily [21]. Therefore, these findings clearly show that PSD is directly linked to the resultant effects of post-stroke hyper-locomotor activity [21,22]. Paolucci et al. [15] support the finding that people suffering from PSD have higher Rivermead Mobility Index (RMI) scores concerning the length of stay in rehabilitation centers due to a lack of proper coordination in motor capabilities. RMI is used to assess functional mobility in gait and balance after a stroke. In most cases, people who have experienced stroke find it difficult to perform regular and routine day-to-day activities such as walking, bathing, sitting, or turning in bed without aid. The association of the challenges faced with PSD and motor activity is not surprising since the disorder causes physical stress that leads to motor impairments.

Cramer [23] argues that motor disorders are associated with reduced quality of life in eighty-two percent of patients. For instance, within six months after stroke, about sixty-five percent of affected individuals face difficulties in effectively incorporating paretic hands into daily activities. Their subjective well-being is also significantly affected because lower extremity motor status increases their disability level. Further findings from Cramer [23] point out that only thirty-seven percent of patients with stroke are in a position to walk after the first week following the stroke. Recovery is often slow because of lower gait improvement, though medications can help.

Severe hyper-locomotor deficit, which is among the risk factors of PSD, may lead to a high degree of disability for a patient if prevention and treatment strategies are not applied early enough. Most of the hyper-locomotive activities that occur after stroke are usually abnormal involuntary movements, which can appear in an acute or delayed sequel. However, the frequency of hyper-locomotive activities and disorders is unclear because the time course for their development varies among individuals. The brain’s basal ganglia are responsible for regulating post-stroke depressive moods and post-stroke movement disorders, including hyper-locomotor activity [10]. When the brain area is affected by stroke, a lower basal ganglia output occurs, thereby causing disinhibition of thalamocortical systems. As a result, the cortical motor areas are released, causing suppressed movements.

Because it is a neurological condition, strokes impact activity. The reduction of the body’s energy efficiency due to the functional damage to the nervous system decreases upper limb and lower limb functions. Thus, it is difficult for one to have postural control of the limb functions and perform daily activities such as dressing post-stroke [24]. In addition, dysfunction in the neuronal circuitry and alterations in basal metabolism of PSD patients impair their motor capabilities. The resultant adverse effect inhibits the functionality of the spinal and locomotor networks, which makes it impossible for an individual to control movement and motion. The arising weakness and spasticity make the affected individuals have stereotypical hemiplegic gait [25]. Therefore, they have low or poor biomechanical performance despite the body using a lot of energy to function healthily [24]. The lower limb muscles are often organized in synergy modules during walking, which enables a stable stance weight, and swing phases. However, the number of synergy modules decreases after stroke, leading to significant changes in the pathways used by chemical messengers. As a result, the affected individuals end up facing challenges in adjusting their walking speed due to reduced fluid movements.

Impairment of cellular mechanisms post-stroke affects the functioning of neuronal tissues and networks and also causes stress for the patients. Stroke survivors also tend to have higher stretch reflex excitability in contralateral limbs compared to healthy individuals. The condition, known as spasticity, is characterized by hyperreflexia and is caused by increased spinal motoneuron excitability [25]. This type of stroke-induced spasticity is challenging to reconcile since it makes the reticulospinal tune motor command system project bilaterally to the spinal column more often. About a third of stroke survivors develop exaggerated stretch reflex responses compared to intact neurological persons.

In most cases, post-stroke hyper-locomotor activity in patients is usually characterized by abnormal involuntary movements (AIMs), such as chorea, tremor, dystonia, parkinsonism, and myoclonus. Research conducted by Alarcon et al. [26] established that a majority of patients develop AIMs up to one year after a stroke. These movements tend to persist over time despite the functional recovery of motor deficits. Chorea is the most frequent class of AIMs, mainly affecting the elderly. It leads to an involuntary movement that attacks the individuals suddenly, briefly, and in a non-repetitive manner. On the other hand, dystonia prevalent among young patients is characterized by sustained muscular contractions. Brain damage in the early stages of life tends to lead to dystonia compared to other AIMs. The affected experience is twisted and repetitive movements that often lead to abnormal posturing. Tremors make one have rhythmic oscillation of body parts. Chorea, dystonia, and tremor can affect a single body part or two or more adjacent or non-adjacent parts, be generalized, or attack the ipsilateral arm and leg [26]. Individuals who are suffering from Parkinsonism exhibit symptoms such as muscular rigidity, postural instability, and rest tremors. The evidence, therefore, suggests that depression and motor impairments are associated, and remain the most prevalent deficits after a stroke.

## 4. Pathogenesis of PSD

**Figure 1:**
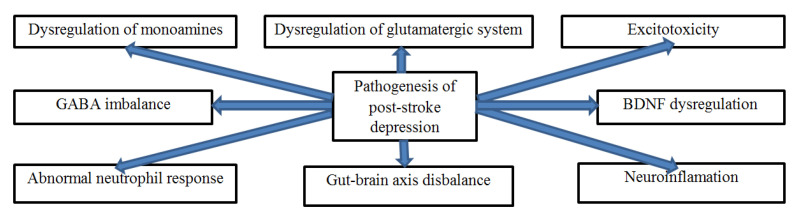
Schematic of the pathogenesis of PSD. The multimodal pathogenesis of PSD can be best understood by examining the roles of the monoamine system, glutamatergic system, excitotoxicity, gut-brain axis, neuroinflammation, and abnormal neutrophilic response.

The pathogenesis of PSD is complex since it involves unique neurobiological mechanisms that include the interaction of neuroanatomical, neuron, and biochemical factors and neurogenesis (Figure 1). Several hypotheses, including lesion location, biogenic amines, inflammation of cytokines, and gene polymorphism, have also been formulated to explain the pathophysiology of PSD [5]. Various research indicates that large lesions in brain areas, such as the left frontal lobe and basal ganglia, can cause disruptions in the critical pathways responsible for controlling moods, leading to depression [27]. The lesion location hypothesis further indicates that depression severity associated with the left frontal lobe is stronger in the first six months after a stroke [5]. Consequently, the buildup of silent cerebral lesions can also alter the normal and healthy functioning of the pathways of several neurotransmitters, including monoamines. Thus, it causes depressive symptoms. However, some studies suggest that its pathogenesis might also be associated with behavioral and social factors. Lai and McCullough [28] argue that the severity of PSD symptoms often goes unnoticed, underdiagnosed, or underestimated by physicians and family members because of the complexity of its pathogenesis.

Severe strokes cause brain inflammation, thereby elevating inflammatory cytokines responsible for regulating the neuroendocrine stress system [29]. Affected inflammatory cytokines may end up suppressing neurotrophic factors in the brain, leading to depressive symptoms.

The immune system of PSD-affected individuals is often activated after a stroke, producing more cytokines. The exponential increase of cytokine molecules increases glutamate excitotoxicity, thereby leading to the enlargement of infarctions and deaths of cells in vital areas of the body [27]. Moreover, resultant stress due to the effects of a stroke induces hypercortisolism, causing a decrease in the overall number of intracellular serotonin transporters. Serotonin is responsible for the essential biochemical changes in brain fluids. If the neurotransmitter (serotonin) is disrupted, the normal function of the CNS is affected and has a significant impact on mood [4]. For example, brain injury survivors experience difficulty feeling positive emotions [30].

**Figure 2:**
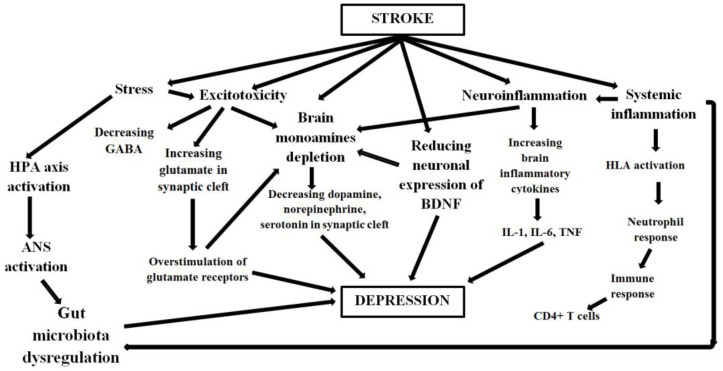
The complex pathogenesis of PSD including different molecular, inflammatory, and physiological mechanisms. (HPA—hypothalamic-pituitary-adrenal axis, ANS—autonomic nervous system, GABA—gamma amino-butyric acid, BDNF—brain-derived neurotrophic factor, HLA—human leukocyte antigen complex).

The multimodal pathogenesis of PSD can be best understood by examining the roles of the monoamine system, glutamatergic system, excitotoxicity, gut-brain axis, neuroinflammation, and abnormal neutrophilic response (Figure 2). It is also essential to analyze the critical roles played by human leucocyte antigen (HLA) dysregulation and other molecular mechanisms.

### 4.1. The Role of the Monoamine System

The contributions of the monoamine system in the pathophysiology of PSD are best analyzed by the biogenic amines hypothesis proposed by Robinson and Bloom [31]. Neurotransmitters, norepinephrine (NE), serotonin (5-HT), and dopamine (DA) are the biogenic amines that form the monoamine system, that are re-uptake by dopamine transporters (DAT), norepinephrine transporters (NET), serotonin transporters (SERT), respectively [4,31]. According to the theory, ischemic lesions in the brain cause unprecedented interruptions of the biogenic amine-containing axons, which ascend from the brainstem nuclei as it extends to the cerebral cortex [5]. The resultant dysfunctions lead to a decline in the release of 5-HT and NE in the basal ganglia and the limb structures found in the temporal and frontal lobes. The disturbances caused by the lesions found in the anterior parts of the frontal lobes prevent monoaminergic bundles from ascending in a normal and healthy pattern.

**Figure 3:**
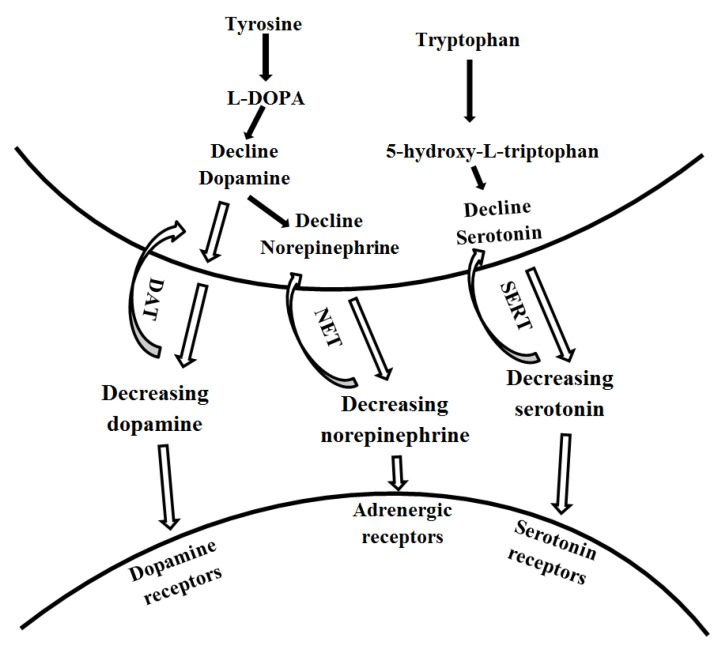
Role of monoamine system in the pathophysiology of post-stroke depression. A schematic mechanism of decreasing concentration of monoamines in the synaptic cleft. (DAT—Dopamine transporter, NET—norepinephrine transporter, SERT—serotonin transporter).

The monoamine system enhances the overall coordination of bodily function. The primary role of NE, 5-HT, and DA is transmitting information between the nerve cells and effector cells [4]. Therefore, when the three monoamine transmitters defect from their pathways, incoordination between body function and the nervous system occurs. Thus, it becomes challenging for biogenic amines to regulate emotions [5]. Thus, a decline in monoamine synthesis that occurs after a stroke causes enzyme inhibition, leading to a decreased concentration of monoamines in a presynaptic terminal as a result of the decline in the concentration of monoamines in the synaptic cleft, respectively (Figure 3). The reducing the regulation of secretion of hormones dealing with emotions and mood. Changes in transmission of monoamines such as serotonin may happen in the early phases of a stroke.

### 4.2. The Role of the Glutamatergic System

The glutamatergic system comprises glutamate receptors located in the brain, spinal cord, neurons, and glia. As a major excitatory neurotransmitter in the nervous system, it has various normal physiological functions. Given that glutamate is an amino acid neurotransmitter; its pathways are linked to several other neurotransmitter pathways. Therefore, any dysfunction or disruption of glutamate can cause profound injurious effects on an individual. Research shows that more than thirty proteins in the glutamate modulate neuronal excitability that is tightly controlled through various measures at the synapse [32]. Depression or mental uneasiness can destabilize the regulatory system, which causes an excess release of glutamate. Such an overproduction can induce hyperexcitability in post-synaptic neurons to an extent where it can lead to cytotoxicity (cell death).

The excessive release of glutamate can also lead to stress and several forms of neurodegenerative diseases. According to Li et al. [33] dysfunctions in glutamatergic neurotransmission can also cause the occurrence of many psychiatric conditions and abnormalities, including major depressive disorder. As the most abundant excitatory neurotransmitter in the brain, glutamate performs critical and multiple functions, including sleep-wake cycle management and acting as an energy source for cells, chemical messenger, and pain signaler [34]. It also stimulates neurons in the brain, thereby enabling the sending of messages, and making gamma-aminobutyric acid (GABA). GABA is responsible for calming the body through its involvement in sleep, relaxation, and anxiety regulation.

Glutamate is produced by nerve cells and later stored in synaptic vesicles containing a high concentration of neurotransmitter molecules. Chemical messages or signals in the brain traveling along the nerve cell pathways initiate the release of glutamate into a synapse (fluid-filled space found between cells in the nervous system). It then binds with specific message-receiving receptors from one nerve cell to the next until the intended destination is reached. Glutamate is a significant presence in the brain because it can bind, work, or partner with four receptors [34]. The ionotropic glutamate receptors (iGluRs) and metabotropic glutamate receptors (mGluRs) are some of the receptors that decode the functional diversity of the glutamatergic system [35]. Therefore, glutamate’s ability to stimulate and communicate with various nervous cells grants it control over ninety percent of excitatory functions in the brain [36]. Various research findings show that interference of glutamate uptake from the synapse leads to reduced sensitivity to reward, which is a primary sign and symptom of depression. Due to its functional significance, it is essential for it to be tightly regulated [37]. Dysregulation can lead to high concentrations of extracellular and extrasynaptic glutamate, leading to cellular damage. The resultant abnormalities can also damage nerve health and communication.

Any activity that inhibits the ability of glutamate to perform its excitatory function can typically lead to various forms of psychological disorders, including depression. In their research, Feng et al. [27] found that glutamate is directly involved in the pathogenesis of PSD. An overload of glutamate release might cause adverse effects since it alters normal brain functioning. The biochemical profile of the prefrontal regions of stroke patients with PSD tends to show a heightened glutamine/creatinine ratio compared to patients who are not suffering from depression [38]. However, no studies have yet identified the mechanisms of how increased levels of glutamate are associated with stroke onset.

**Figure 4:**
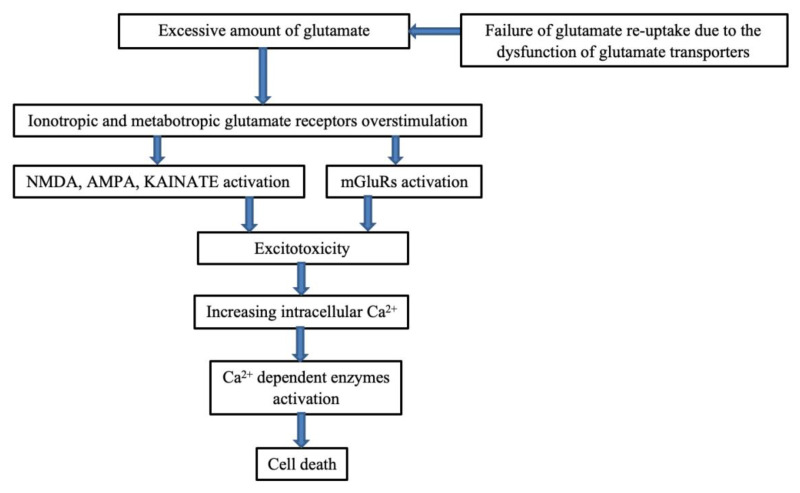
Glutamate excitotoxicity. A schematic of the mechanism of cell death triggered by excessive glutamate release.

As a non-essential amino acid, glutamate compositions account for more than 55 percent of all neuro-mediators’ activities in the brain. With a stroke, glutamate concentrations in the cerebrospinal fluid (CSF) and extracerebral fluid increase more than three hundredfold [9]. Such an increase causes damage to neurons since glutamate can spread extensively to areas beyond the infarcted tissue. Feng et al. [27] report that the size of the infarction is a vital characteristic that can determine the severity of PSD. Thus, the increment of glutamate may disrupt the blood supply in the necrotic tissue in the brain (cerebral infarct), causing large infarctions. An excessive release of glutamate stimulates its receptors, which can not only lead to cell swelling but also cause apoptosis and the eventual death of neurons [9] through the activation of calcium-dependent enzymes (Figure 4). On the other hand, the excitatory amino acid transporter system also becomes injured, so excessive glutamate cannot be removed from the synaptic cleft. The neurological deficits caused by large infarctions can lead to severe impairment of vital areas that regulate emotional behavior and biochemical change. As well as lesion location, lesion size also acts as a critical social-psychological factor in the early-onset of PSD

Alteration of plasma glutamate levels has been primarily associated with the occurrence of depressive symptoms [39]. Chang et al. [39] established that most depressed individuals had elevated glutamate content in the plasma compared to healthy subjects. Therefore, plasma glutamate acts as a robust biological marker of the risk of developing PSD after the third month following a stroke. Additionally, depressed people tend to have higher infarct volumes, increased functional disability, and severe strokes, which are known risk factors for PSD.

Establishing the localization of stroke and PSD has been contentious, given that both lesion location and size act as huge determinants of the condition [39]. Glutamate assumes a mediating role in neuronal degeneration during ischemia, causing a cellular overload of calcium. The resultant adverse effect of such an overabundance is a breakdown of cellular structures and necrosis. For purposes of obtaining adequate and reliable results, Cheng et al. [39] assessed glutamate plasma levels without previous acid or base treatment to avoid potential interference. The study established the existence of a strong relationship between the levels of glutamate of patients at admission and the development of PSD. This study corroborated findings that established a strong association between the early-onset PSD and a low plasma glutamate level [40].

The glutamatergic system influences the neurobiology of depression. Several studies have shown that the prefrontal cortex and temporal cortex of PSD patients have protein of glutamate receptors, which is an indicator of glutamatergic dysfunction [41]. The receptors responsible for the modulation of mood and other emotional aspects related to depression include the ionotropic (N-methyl-D-aspartate [NMDA], α-amino-3-hydroxy-5-methyl-4-isoxazole propionic acid [AMPA]), kainate receptors, and metabotropic (mGluR) glutamate receptors [41]. Glutamate alterations increase the serum levels. However, patients with inherent conditions such as refractory affective disorder tend to have lower glutamate levels in the CSF [40]. It has been conclusively shown that the neurological underpinnings of glutamate dysfunctions contribute to the pathophysiology of mood disorders, including post-stroke depression.

### 4.3. The Role of Excitotoxicity

Excitotoxicity is a process involving the degeneration of nerve cells and neural death. It results when a large volume of the excitatory amino acid glutamate is produced and allowed to accumulate in the spaces between cells (Figure 4). Strokes can cause energy failure, thereby limiting the ability of adenosine triphosphate (ATP) to maintain a normal membrane ionic gradient [42]. ATP is an energy currency in the cell, acting as a neurotransmitter in the peripheral system and CNS. It transmits signals in neuronal and non-neuronal tissues and between glial cells and neurons. When the glutamate receptors are overstimulated, disruption of ionic homeostasis occurs since ATP becomes depleted. The sodium and calcium influx that happens during the activity affects all subcellular compartments [43]. Significant changes occur in the cytosol, endoplasmic reticulum (ER), nucleus, and mitochondria. Glutamate receptor (AMPA-kainate and NMDA) inducement can impair cells, especially when the brain is subjected to stress.

Excitotoxicity is a significant contributor to the pathogenesis of brain injury as a result of a stroke and as an agent of human illnesses. The toxic actions of excitatory neurotransmitters such as glutamate can prompt neurotoxicity that can culminate in the loss of neuronal function [44]. Glial elements such as astrocytes are tasked with the duty of managing alterations between normal and healthy physiological processes and excitotoxicity. It regulates and clears excess glutamate available in the synaptic cleft. Excitotoxicity is triggered when there are disruptions in glutamate and calcium metabolism, damage to glutamate transporters, and impairment of glutamate receptors [44]. Other cellular mechanisms that can lead to excitotoxicity include oxidative stress, physical neuronal damage, and mitochondria malfunctioning [45]. Sustained movement of calcium into the chemical messengers causes several adverse consequences, including limiting the reabsorption that contributes to neuronal loss.

Excitotoxicity phenomena also play a central role in the pathogenesis of several neurodegenerative diseases, including depressive disorder [44]. Even though glutamate does not directly kill cells, the harmful actions resulting from the prolonged activation of NNDA or calcium-permeable AMPA receptors set forth a cascade of neurotoxicity [45]. The cause of early brain injury can be best explained by glutamate excitotoxicity. As mentioned, when glutamate is released in excess, neuronal hyperexcitation results, thereby instigating post-stroke torrents that can lead to secondary neuronal damage [46]. On the other hand, the spreading depolarization (SD) theory asserts that the injury to neurons is sometimes evoked by metabolic stress rather than by excessive glutamate release.

The interference of the electrochemical membrane and neuronal swelling in the grey matter of the grain is primarily caused by the SD mechanism. The process causes various diseases and conditions, including concussion, intracerebral hemorrhage, ischemic stroke, and migraine-associated aura [46]. The brain is vulnerable to ischemic damage that can continue progressively for months. Since it is linked to glutamate, excitotoxicity in stroke can spread depression and other neurodegenerative diseases. Lai et al. [47] argue that glutamate-mediated is the primary cause of neuronal death, thereby leading to harmful effects among patients after stroke. Belov Kirdajova et al. [48] assert that the pathophysiology of several neurological disorders, including post-stroke depression, follow a common deadly excitotoxicity pathway.

Present studies show that oxidative stress has a considerable role in the pathophysiology of various forms of depression, including PSD. Stress is an excitotoxic event that occurs when glutamate receptors are massively activated, and it causes an imbalance in the brain, increasing the production of free radicals to toxic levels. The free radicals, consisting of atoms and molecules capable of independent existence, are usually highly reactive to the extent that they can oxidize and impair proteins [49]. Moreover, they can initiate reactions such as mitochondrial dysfunction that can lead to neuron and excitotoxic death in the brain after a stroke. Some of the radicals whose synthesis is triggered by excitotoxicity include superoxide radicals and hydroxyl radicals. The neuronal loss in the focal areas of the brain disrupts the transmission of sensory input, including the regulation of mood and emotions [50]. Such dysfunction is associated with severe mental illnesses after a stroke, including PSD.

A rise in the volume of serum glutamate levels can lead to a poor prognosis and increase the severity of PSD. The abnormal neurotropic responses caused by glutamate-mediated excitotoxicity increase due to the cytokines produced after stroke. The following complex processes, including the enlargement of cerebral infarctions due to neuronal death and hypercortisolism, can induce stress [51]. Moreover, inflammation that often occurs post-stroke reduces intercellular serotonin transporters, thus increasing the risk of depression and associated symptoms. These neurobiological processes are only experienced by individuals who suffer from PSD.

### 4.4. The Role of the Gut-Brain Axis

The gut-brain axis (GBA), also known as the enteric nervous system (ENS), comprises millions of nerve cells that line the gastrointestinal tract from the esophagus to the rectum. Its primary role is to communicate with the “big brain” to control activities such as the digestion of food from the initial process of swallowing to the elimination of unwanted food particles from the body [52].

It can also cause emotional shifts, especially among individuals struggling with digestive and bowel problems such as constipation, irritable bowel syndrome (IBS), diarrhea, and stomach upset. Moreover, researchers have established that the GBA contributes to motivation, anxiety, and depression. According to Carabotti et al. [53], the gut microbiota influences these interactions since the GBA can send signals to the CNS, thereby triggering the resultant mood changes.

The human gut contains several microorganisms on its lining, which are physiologically essential to the digestive system. These microorganisms enact fermentation of undigested food content to produce energy and nutrients and sustain the body’s immune system [54]. Apart from common digestive and immune functions, microorganisms participate in brain functionality and behavior.

The microbiome is an essential part of body organs through its role in major physiological activities. A healthy individual is supposed to have a broad set of bacteria that has several microbial genes that aid in the physiological processes [55]. The GBA is made up of the central system, the enteric nervous system, and the digestive system, which operate in coordination [56]. Combined, the collective system contains the microbiota, which performs several roles: hormonal secretion, gut motility, production of acid, bicarbonates, mucus in the nervous system, digestion, growth, and immunity in the gut system. A microbiota includes all microbial classes. It primarily comprises bacteria, archaea, eukaryotes, and viruses that can be beneficial or disastrous to the functioning of the body systems. One of the vital microbiomes is the intestinal microbiome which has a close connection with the CNS. For stress-related health conditions, microbiome administration through probiotics has exhibited desirable results and efficacy.

IBS is associated with stress-related mental illnesses. Studies involving germ-free rodents, antibiotics, probiotics, and gastrointestinal tract infections have suggested that intestinal microorganisms participate in brain regulation, behaviors, and stress response and management through the microbiota-gut-brain axis (MGB) [57]. The system facilitates coordination with the CNS through direct and indirect channels. The MGB forms part of the physiological network that comprises the endocrine system, immune system, autonomic nervous system, and enteric nervous system. In the CNS, MGB produces bacterial metabolites with the help of tryptophan metabolism to trigger the hypothalamic-pituitary-adrenal axis. These triggers and nervous activities promote CNS development.

The GBA links the cognitive and emotional centers of the brain with peripheral intestinal functions, creating bidirectional communication. The CNS, brain and spinal cord, automatic nervous system (ANS), the hypothalamic-pituitary-adrenal (HPA) axis, and the ENS must all interact and work together for effective communication [53]. The afferent signals originating from the lumen are transmitted through the enteric, spinal, and vagal pathways to the CNS and the intestinal wall lining. The HPA axis, which is part of the limbic system, coordinates adaptive responses to any type. Alterations in the gut microbial can cause dysbiosis, usually associated with diseases of gastrointestinal and distal organs [58]. Other disease-causing organisms that might affect the gut, such as intestinal bacteria, can also negatively affect the normal physiology of the CNS. These dysfunctions limit the ability of the GBA to control memory and emotional responses efficiently.

When dysbiosis occurs, the bidirectional communication pathways are dysregulated. As a result, the inflammation of the neurons happens, and the permeability of the blood-brain barrier is altered. However, besides dysbiosis, mechanisms from neuroinflammatory conditions such as depressive disorders and Alzheimer’s can cause the inflammation of the pathways [58]. Additionally, environmental stress can also cause adverse disruptions to the normal functions of GBA [53]. According to Carabotti et al. [53], stressors induce the secretion of corticotropin-releasing factor from the hypothalamus, thereby activating the system. Moreover, dysbiosis can also occur in functional gastrointestinal disorders. The disruptions caused by these disorders are linked to GBA and can lead to mood disorders. The contribution of gut microbiota not only ends with neuroinflammatory and psychiatric conditions but also extends toward brain development. Several beneficial bacteria in the gut interact mutually to maintain the health of intestinal organs and the whole body. However, environmental factors can alter the composition of these bacteria and the microbiota, causing alterations [58]. For instance, the absence of Fusobacteria will lead to a shorter relapse time in patients with multiple sclerosis.

The primary stress hormone cortisol significantly affects the normal functioning of vital body organs, such as the brain. As a result, the neural and hormonal lines of communication are forced to combine and work together to enable the brain to carry out its purpose with minimal resistance. Thus, cortisol manages to efficiently influence the activities of intestinal effector cells, enterochromaffin cells, and interstitial cells of Cajal that are controlled by gut microbiota [53].

The gut and brain are connected through neuronal messengers, the vagus nerve, and the nervous system. The neurotransmitters produced by the brain are similar to those synthesized in the gut. The gut also secretes GABA, which is responsible for controlling feelings of fear and anxiety [59]. Consequently, microbes found in the gut also affect the brain by producing chemicals known as short-chain fatty acids (SCFA). SFCA, which includes butyrate, propionate, and acetate, affects brain function by reducing appetite and food intake and, at times, forming the barrier between the brain and the blood. Since the gut and the brain work in synchrony and have reciprocal communication, applying any intervention or treatment to one can help the other [60].

### 4.5. The Role of Neuroinflammation

Neuroinflammation plays an essential role in the pathogenic mechanisms of PSD, though the molecular and cellular mechanisms of the process are complex (Figure 2). They involve astrocytes, microglia (sentinel cells of innate immunity in the brain), cytokines (tumor necrosis factor-alpha interleukin 1, interleukin 6), microRNAs, and monocyte-derived macrophages [6]. Strokes, which usually arise due to the blockage or rupture of the blood vessels, prevent the free flow of oxygen and glucose in the brain tissue, disrupting the production of adenosine triphosphate (ATP). According to Wen et al. [61], the low concentration and depletion of ATP lead to cell damage and slow death. The dead cells then release danger-associated molecular patterns that are recognized by toll-like receptors (TLR) (found on the surface of microglia) and eventually binding together. The binding instigates signaling that invokes the localized secretion of inflammatory cytokines IL-1β [61]. The production of cytokines is an automatic response that occurs when cellular damage or invasive pathogens are caused by bodily injury or infection.

The extent of brain injury after stroke largely depends on the magnitude of the inflammatory series of events driven by the cytokines. Neutrophils and macrophages are other inflammatory cells that are activated in the event of such an injury [6]. Data from Wen et al. [61] indicate that the resulting inflammation causes dysfunction of hypothalamic orexin-secreting neurons, lowering the production and regulation activity of monoamine neurotransmitters. Moreover, the immune response activated by the cells modulates synaptic plasticity changes during the stroke rehabilitation process [6]. Stroke-induced immune reactions caused by astrocytes, inflammatory cells, and ambient microglia are associated with the onset of PSD and its resultant adverse effects.

### 4.6. The Role of Abnormal Neutrophilic Response

Neutrophils are effector cells that maintain the immune system’s strength by trapping and killing invading pathogens in the body. They are regarded as the first line of defense and the body’s most abundant leukocytes. They perform their role through phagocytosis, intracellular degradation, releasing granules, and forming neutrophil extracellular traps [62]. However, the function of neutrophils is not limited as they have broad phenotypic heterogeneity and functional versatility. Besides their primary function of trapping and destroying disease-causing organisms, these cells can also respond by producing cytokines and other inflammatory factors [62]. The anti-inflammatory cytokine is responsible for regulating inflammation and the immune system. Abnormal neurotropic response limits the production of cytokines, making it difficult to modulate inflammation that may cause cell death and dysfunction of mood-regulating neurotransmitters. The neutrophil-to-lymphocyte (NLR) ratio determines the responses initiated by neutrophils and is used as an indicator of inflammation, which is associated with strokes and the development of PSD [63]. Hu et al. found that PSD patients have increased levels of NLR compared to normal healthy individuals [63]. A higher NLR increases PSD risk since it is associated with a poor prognosis for ischemic disorders (Figure 2).

An increase in the concentration of neutrophils during brain injury may damage endothelial cells, causing chronic inflammation in the process. As the high number of neutrophils reaches the injury site, they coincide with the pro-inflammatory cytokines and the NLR, further amplifying the immune cascade [63]. The overproduction of cytokines by the HPA axis affects the ability of chemical messengers to regulate physical pain, blood pressure, mood, and depression. Cellular dysfunction occurs as inflammation changes the functionality of the intracranial neuroendocrine. The production and secretion of monoamine neurotransmitters are also reduced in the process, initiating the development of PSD.

### 4.7. The Role of HLA Dysregulation

The human leucocyte antigen (HLA) helps regulate immune responses in the human body, and its immunological function of HLA is controlled by genes [64]. HLA are classified as significant histocompatibility complexes (MHCs) due to the vital role they play. However, possible mutations of the MHC can cause HLA dysregulation, increasing the chances of autoimmune diseases [65]. The molecules regulate the immune response by acting as presenters of self and foreign peptides and antigens to T cell receptors responsible for initiating tolerance in case of an attack.

Research findings from Tubb et al. [7] show that dysregulation of immune function is linked to infectious diseases and psychiatric disorders, such as depression. Clinical studies show an inherited biological basis of depression, with additive genetic factors accounting for variation in susceptibility among the affected individuals. There are about 269 genes considered to be underlying contributors to depressive conditions such as PSD [7]. The regulatory mechanism of HLA constitutes the primary line of defense against infection as it detects new antigens in the body and produces corresponding antigens. The HLA-alleles respond against invading pathogens in the immune system by activating CD4+ T cells and inducing the secretion of cytokines [66]. In turn, the innate immune system initiates acute inflammation as a defensive mechanism following the release of cytokines. Additionally, systemic inflammation can lead to neuroinflammation (Figure 2) that impairs the normal and healthy functioning of neurotransmitters responsible for regulating mood and emotions. As a result, affected individuals exhibit symptoms such as persistently low mood, poor concentration, sleeplessness, and hopelessness, which are all linked to PSD.

### 4.8. Other Molecular Mechanisms

Molecular mechanisms, such as brain-derived neurotropic factor (BDNF), also play a significant role in the development of post-stroke depression (Figure 2). Even though the details of the influence of the workings of BDNF in the occurrence of PSD are still little understood, research shows that strokes may affect the expression of BDNF in the brain. Zhang and Liao [67] assert that patients have lower serum levels of the neurotropic factor in the hippocampus, increasing their vulnerability to adverse depressive symptoms of PSD. Research evidence from Shan et al. [67] also shows that the neurotropic factor directly interacts with neurotransmitters such as the serotonergic and glutamatergic systems associated with the underlying mechanisms of PSD.

The link between BDNF and PSD has also been supported by research in the field of genetics. It has been established that higher BDNF methylation status reduces neuronal expression of BDNF. DNA methylation is involved in a number of critical bodily functions. When its balance is disrupted, it can cause several health problems, including depression [8]. Li et al. [8] also supported the correlation between DNA methylation, BDNF, and depression, ascertaining that BDNF and nuclear receptor subfamily 3, group C, member 1 (NR3C1) gene methylation levels are linked to depressive symptoms. Thus, BDNF is significantly and independently associated with the pathophysiology and incidence of PSD.

Therefore, any alteration in the serum level of BDNF decreases the neuroprotective functions in brain pathology responsible for regulating mood and emotions. Reduced levels of BDNF also inhibit its ability to induce antiapoptotic mechanisms in the brain injury caused by stroke, thereby making it challenging to impede cell death [67]. The low expression of the brain-derived neurotropic factor might cause PSD since it decreases neurogenesis in the hippocampus [68].

## 5. Current Drug Research

**Figure 5:**
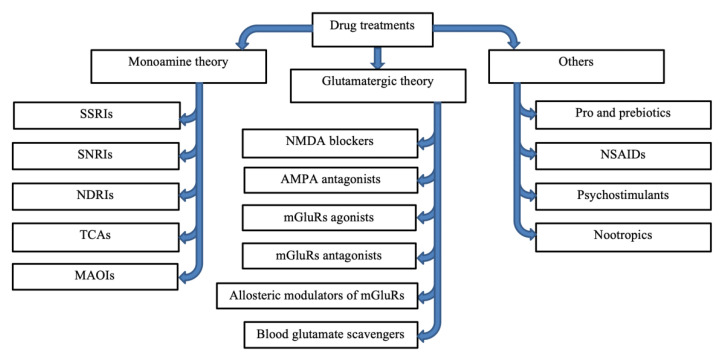
Approaches to drug treatment of PSD, categorized by their utilization of monoamine theory, the glutamatergic theory, or others.

The complex nature of PSD mechanisms and associated conditions has made it a challenging task for medical providers and scientists to design physiologically based prevention and treatment. However, a wide array of scientific research has aided in the development of several categories of drugs that have proved to be clinically efficient and effective in preventing the treatment of PSD. The pathophysiology of PSD is multifactorial since it involves a combination of ischemia-induced neurological impairments [69]. Thus, most drugs are manufactured and developed to counter the effects of damages caused by factors involved in the pathogenesis of post-stroke depression (Figure 5).

### 5.1. Research on Drugs That Are Based on Monoamine Theory

Based on the monoamine theory, PSD’s pathophysiology causes significant depletion of serotonin, norepinephrine, and dopamine neurotransmitter levels in the CNS. Gu et al. [70] note that the disruption of neurotransmission induces the release of proinflammatory cytokines, which can cause abrupt dysfunction of cortical circuits responsible for mood regulation and monoamine production. As a result, several types of antidepressant drugs have been developed based on the monoamine hypothesis to elevate the levels of these neurotransmitters in the brain to alleviate symptoms of depression, shorten recovery times, and improve the overall quality of life.

The monoamine transmitters that are involved in emotion regulation and play a vital role in the treatment of PSD include 5-hydroxytryptamine, which regulates disgust; dopamine, which regulates happiness; and norepinephrine, which regulates fear and anger [70]. The main drugs that have been approved by the Food and Drug Administration for the treatment of PSD based on the monoamine theory include tricyclic antidepressants and selective serotonin reuptake inhibitors (SSRIs), and serotonin-norepinephrine reuptake inhibitors (SNRIs) [71]. Other drugs that have proved to be effective are norepinephrine and dopamine reuptake inhibitors (NDRIs) and monoamine oxidase inhibitors (MAOIs).

#### 5.1.1. SSRIs

SSRIs and antidepressants comprise drugs such as fluoxetine, paroxetine, fluvoxamine, sertraline, citalopram, and escitalopram. Ambrosi et al. [67] propose that SSRIs are highly effective in combating PSD in that they allow affected individuals to participate in rehabilitation efforts with limited struggle. Using SSRI drugs to treat PSD help alleviate several adverse effects of the disorder, such as headache, insomnia, and gastrointestinal functions [70]. PSD causes disinhibition of neural circuits and exaggerates neural excitation, disrupting the balance in the cerebral cortex needed for motor learning and synaptic plasticity [71]. The administration of SSRIs helps normalize the overexcitation and inhibitory signaling capable of causing post-stroke neural plasticity impairment and regeneration. As a result, SSRIs can promote recovery in PSD patients since it augments cellular defense pathways damaged during ischemic injury [71]. The drugs also enhance the regulation of cerebral blood flow through the direct activity of vasoactive monoamines, limiting neuronal death in the peri-infarct area.

SSRIs can also help promote recovery from PSD through a number of pleiotropic activities, such as neuroprotection and simulation of spontaneous neurogenesis. Its neuroprotection role is shown through drugs such as fluoxetine which prevents inflammation in the post-ischemic brain of patients [69]. Together with sertraline, fluoxetine also helps improve the autoregulation of cerebral blood flow (CBF) and vascular tone independently of nitric oxide synthase (NOS)–related pathways. Most research findings show that SSRIs are more effective in improving outcomes for individuals in less than twelve months after a stroke compared to placebo [72]. Most people also tolerate antidepressants because they have benign, transient, and rare side effects.

However, for maximum benefit, SSRIs should be administered early. In their study, Zhou et al. [73] established that antidepressants are more effective in the treatment of PSD rather than in its prevention. SSRIs achieve their intended purpose by enabling the redistribution of the motor cortex in the post-stroke population. Depressed patients also end up experiencing reduced dependency ninety days after the administration of the drugs [74]. Each of the classes of drugs under SSRIs has secondary pharmacological properties and functions other than serotonin transporter (SERT) blockade. For instance, paroxetine has mild anticholinergic action, while sertraline can effectively perform the role of dopamine transporter binding [74]. The SSRI antidepressants also differ from other PSD treatment drugs in that they can enable drug-drug interactions since they can cause the inhibition of cytochrome-P450 isoforms.

Despite its effectiveness, there are some concerns about the potential risks of SSRIs to PSD patients. There have been arguments that the antidepressants may have severe side effects for some users and, at times, induce depressive symptoms that it was intended to prevent or treat. For instance, a study by Mortensen et al. [75] shows that SSRIs can cause insomnia, intracerebral bleeding, and sexual dysfunction. A possibility of higher mortality of people suffering from stroke using SSRIs has been demonstrated independently of depression at three months, due to the increased bleeding risk [76]. However, the results are inconclusive and warrant further research. Stakeholders, including medical professionals, should carefully and closely monitor patients to substantiate the dangers of SSRIs and clinical outcomes.

In addition, Zhou et al. [73] performed a meta-analysis to determine the efficacy of SSRIs for restoring or improving functional independence and preventing depression in the beginning post-stroke stage. A total of 5370 patients were enrolled in the study. The results showed that SSRI therapy was most effective in preventing PSD when used in the initial stages of the condition. Similarly, Sun et al. [77] integrated direct and indirect evidence to compare the efficacy and acceptability of SSRI antidepressants in treating PSD, using several randomized controlled trials (RCTs) and meta-analyses (Table 1). The results established that the acceptability scores for doxepin, citalopram, and fluoxetine were higher than paroxetine. The researchers concluded that paroxetine was the preferred drug at the onset of acute treatment for PSD.

Li and Zhang [78] also performed a meta-analysis to investigate the comparative efficacy of citalopram, escitalopram, venlafaxine, paroxetine, duloxetine, amitriptyline, doxepin, sertraline, and mirtazapine in the treatment of patients with PSD. Based on data analysis from 51 RCTs, escitalopram was the most favorable, while amitriptyline scored the lowest at two weeks. Citalopram was more effective than the rest of the SSRIs at four weeks, while mirtazapine had the highest response rate among patients after eight weeks.

#### 5.1.2. SNRIs

SNRIs are another class of antidepressants introduced in 1993 and approved by the FDA to help manage and treat post-stroke depression. Desvenlafaxine (Pristiq), venlafaxine, levomilnacipran, and duloxetine (Cymbalta) are commonly used SNRIs [75]. The drugs are given in varying doses depending on the patient and the severity of their conditions. For instance, duloxetine is administered at 60 mg twice daily [79]. Venlafaxine, on the other hand, should be given in doses of both short-and-long-acting formulations. They effectively treat depression by blocking the reabsorption of serotonin and norepinephrine neurotransmitters that communicate between brain cells [80]. Thus, these dual-acting antidepressants manage to keep up the levels of the two chemical messengers. Their concentration increases in the synaptic cleft, thereby causing the stimulation of the descending inhibitory system in the spinal cord [81]. By affecting changes in brain chemistry and communication, antidepressants help relieve symptoms of PSD, such as irritability and sadness. They have a similar mechanism of action as SSRIs since they also enhance serotonergic function.

However, despite their documented efficacy in treating PSD, SNRIs also present adverse side effects to some groups of patients, such as pregnant or breastfeeding women. Those taking these antidepressants during the second half of the pregnancy may experience withdrawal symptoms, including difficulty breathing, tremors, and feeding problems [82]. The fetus can also be affected since SNRIs can pass into breast milk. Thus, it is vital for patients in this category to seek advice from a medical practitioner on the antidepressant that best suits their needs.

General side effects, such as muscle weakness, dizziness, nausea, insomnia, and sleepiness, can affect all patients. Individuals can also experience tiredness, constipation, reduced sexual desire, and difficulty reaching an orgasm or maintaining an erection [80]. Drugs such as venlafaxine can cause cardiac conduction boxes, especially among those persons with cardiac disease [79]. Additionally, those with end-stage renal disease, alcohol disorders, and hepatitis avoid duloxetine since it can lead to severe hepatotoxicity. There is also the risk of gastrointestinal bleeding because SNRIs can inhibit platelet aggregation by disrupting platelet serotonin receptors [83]. Sansone and Sansone [83] also point out that youths under that category of antidepressants have a higher likelihood of entertaining suicidal behavior and ideation.

Despite their risks and side effects, the benefits of SNRIs appear to outweigh the negative impacts. Data from the American Heart Association Stroke Council show that the use of antidepressants has proved effective in treating PSD [84]. Zhang et al. [85] investigated the prophylactic effects of duloxetine, a new SNRI, on PSD symptoms. Ninety-five ischemic stroke patients without depression participated in the RCTs. The participants were divided into the duloxetine group (given doses ranging from 30–90 mg) and the control group (subjected to routine ischemic therapy). The results showed that the use of duloxetine helped prevent minor and major depression in stroke patients and also assisted in rapid rehabilitation from strokes. They concluded that prophylactic use also reduced the incidence of PSD and improved individuals’ quality of life and cognitive function.

Cravello et al. [86] performed an RCT to ascertain the effectiveness of the SNRI venlafaxine in improving symptoms of alexithymia in patients with PSD. Twenty-five patients treated with venlafaxine were assessed for up to 8 weeks. The results showed that these patients had more significant improvement in their alexithymia symptoms than the patients treated with fluoxetine. They concluded that antidepressants such as SNRIs were effective in treating depression and improving emotional awareness in PSD patients. Tsai et al. [87] conducted a meta-analysis to ascertain the effectiveness of milnacipran in preventing PSD. The study enrolled ninety-two patients, and half of them were treated with milnacipran. The results established that milnacipran had a significant advantage in preventing PSD without substantial adverse effects.

#### 5.1.3. Norepinephrine-Dopamine Reuptake Inhibitors (NDRIs)

Norepinephrine-dopamine reuptake inhibitors (NDRIs) have increasingly gained popularity in recent times in the treatment of depression due to their efficacy. They are highly recommended for treating PSD since they have fewer side effects than other common antidepressants, including weight gain or lowered sex drive [88]. They perform their intended function by preventing the reabsorption of norepinephrine and dopamine into the brain [88]. The dysfunction of dopamine and norepinephrine increases the risk of mood disorders such as depression. These two neurotransmitters are responsible for controlling and maintaining alertness and concentration (norepinephrine) and perception of reality, ability to experience pleasure, and motivation (dopamine).

There are various types of NDRIs, but bupropion is the most widely used [88]. Patients can use it alone or with a combination of other antidepressants to treat post-stroke depression. Bupropion is available under the brand names Zyban, Alpezin, and Wellbutrin. However, there are also other NDRIs that are not as widely used, such as Survector (amineptine), Cleofil (difemetorex), Catovit (prolintane), and Phenotropil (Phenylpiracetam). In most cases, antidepressants such as SSRIs and SNRIs are usually tried first before NDRIs [89]. Bupropion is often used as a third line of option if the other drugs have proven to be ineffective in treating or reducing the associated symptoms of PSD. First-line depression medication can fail to help about thirty to forty percent of patients [90]. Therefore, clinicians may be forced to add NDRIs if using just one type of drug has failed.

NDRIs are sometimes referred to as atypical antidepressants since their mechanisms of action significantly differ from other drugs used to treat PSD. By blocking the action of specific transporter proteins such as the norepinephrine transporter, NDRIs can increase the amount of active norepinephrine and dopamine chemical messengers throughout the brain [84]. However, despite their efficacy, these newer forms of first-generation antidepressants also have some mild side effects.

Dry mouth, nausea, insomnia, and anxiety are some of the prevalent side effects of NDRIs. In other unique and rare cases, patients may experience severe side effects such as increased blood pressure, suicidal ideation, vision loss, and seizures [88]. However, most of these effects usually improve after individuals have taken the drugs for a few weeks. However, if effects such as dry mouth persist, the affected individuals should try moistening their mouth by sipping water and cutting down on alcohol, caffeine, and smoking. Nausea can be mitigated by taking medication with food [91]. The drugs can also cause dependency if used for a prolonged period, making it difficult to withdraw [91].

In their research to determine the effectiveness of bupropion, an NDRI, as an antidepressant, Patel et al. [92] did a meta-analysis of 51 studies of bupropion and placebo. Through their investigations, they established that bupropion was effective and highly tolerated, with equivalent effectiveness compared to other antidepressants. Patients using it had low rates of side effects such as sexual dysfunction and weight gain. Liu et al. [93] performed a meta-analysis to determine the efficacy of bupropion hydrochloride sustained-release tablets in treating PSD. The results showed no difference in effectiveness between bupropion and other antidepressants such as fluoxetine.

Sung et al. [94] conducted another study to suggest antidepressant efficacy compared to placebo and the difference in effectiveness between several antidepressants. They conducted a meta-analysis and obtained data from various sources, including clinical practice guidelines for depression. The results showed that NDRIs were weakly recommended for their antidepressant efficacy compared to other drugs such as SNRIs and SSRIs. Patients usually determine effectiveness after two to four weeks of treatment.

#### 5.1.4. Monoamine Oxidase Inhibitors (MAOIs)

Monoamine oxidase inhibitors (MAOIs) are among the first batch of antidepressants to be developed and approved for the treatment and management of depressive symptoms. However, they have been gradually replaced by safer drugs that cause fewer side effects. People suffering from PSD must observe diet restrictions when using MAOIs and avoid certain types of medications or risk life-threatening high blood pressure [95]. Therefore, these medications are only a treatment option when other kinds of PSD treatments have failed. Their mechanisms in treating depression resemble that of other antidepressants such as SSRIs, SNRIs, or NDRIs. They affect chemical messengers (neurotransmitters) that aid in communication between brain cells that are operational in regulating moods.

In PSD, the monoamine enzyme stimulates the removal of norepinephrine, serotonin, and dopamine from the brain, affecting the ability to regulate mood efficiently. Enzyme cleaning of neurotransmitters in the body is only carried out once they have carried out their intended jobs [96]. MAOIs are administered to block the action of this enzyme, thereby increasing the concentration and availability of the three neurotransmitters in the brain [95]. It, therefore, becomes possible for the chemical messengers to affect changes in body cells and circuits that have been impacted by PSD, hence positively alleviating depressive symptoms. The MAO enzyme is of types, A and B. Type A, which is comprised of serotonin and noradrenaline substrates, is distributed in the gut, liver, and placenta [95]. Type B contains phenylethylamine, methylhistamine, and tryptamine substrates and is found in the brain, liver, and platelets. Both categories of MAO can metabolize dopamine and tyramine, thus affecting the ability of the hormones to regulate body functions, including memory and pleasurable reward and motivation.

Commonly used MAOIs approved by the Food and Drug Administration include Isocarboxazid (Marplan), Selegiline (Emsam), Phenelzine (Nardil), and Tranylcypromine (Parnate). The antidepressants are primarily administered orally and using a skin patch. A skin patch is highly recommended since it causes fewer side effects compared to oral administration [95]. However, caution should be observed when administering MAOIs since they have higher drug interactions and drug-food interactions. It reacts with aged foods such as soy sauce, cheese, and cured meat since they have higher levels of tyramine [97]. It is also crucial for PSD patients to avoid eating tofu, dates, raisins, dried fruits, alcohol, and all nuts when taking the medication.

MAOIs also tend to have more severe side effects compared to other antidepressants. Dry mouth, nausea, diarrhea, dizziness, confusion, and insomnia are the most encountered effects of the drugs [95,98]. Some patients may also experience problems such as muscle cramps, weight gain, involuntary muscle jerks, high or low blood pressure, and reduced sexual desire [98]. Those with a history of seizures or epilepsy should avoid taking MAOIs [95]. Furthermore, people with problems such as alcoholism, severe headache, blood vessel disease, heart disease, and kidney disease should keep off MAOIs. Better outcomes while MAOIs can be realized when PSD patients discuss with their doctors their conditions and any possible side effects before taking these antidepressants.

Research on MAOIs has been somewhat inconclusive. Li and Gu [99] probed the efficacy of PSD treatment based on its temporal status through a meta-analysis of 34 articles, many of which use MAOIs as a treatment for PSD. The results showed that MAOIs helped reduce or avoid the occurrence of depression and can be an effective tool for patients with PSD. Thase et al. [100] investigated the effectiveness of MAOIs in treating depression. They conducted four outpatient meta-analyses and compared MAOIs such as phenelzine and isocarboxazid with placebo and TCAs. The findings showed that the MAOIs were less effective than TCA for inpatients. However, they were highly effective in depressed outpatients with atypical features. They concluded that MAOIs should not be recommended to patients with severe depressive symptoms due to a preferential responsivity. Similarly, Qin et al. [101] did a network meta-analysis to investigate the efficacy, acceptability, and tolerance levels of antidepressants. Outcomes among the 949 PSD patients were measured based on the changes in signs and symptoms of depression. A total of nine antidepressants were analyzed, including MAOIs. The findings showed insufficient evidence that MAOIs were more effective and tolerable than placebo and other antidepressants such as SSRIs.

#### 5.1.5. Tricyclic Antidepressants (TCAs)

Tricyclic antidepressants (TCAs) approved to treat depression include amitriptyline, amoxapine, desipramine, imipramine, nortriptyline, protriptyline, doxepin, and trimipramine [102]. TCAs have proved to be efficacious in treating PSD patients. For instance, most individuals treated with nortriptyline usually show a decrease in depressive symptoms and heightened recovery of activities of daily living, with minimal side effects [77]. Off-label drugs such as the cyclic drug clomipramine are also sometimes prescribed for the treatment of PSD [76]. The efficacy of TCAs is equivocal to that of SSRIs. However, their anticholinergic activities and lower threshold for overdose make the antidepressants have significant adverse effects compared to those of SSRIs. As a result, they are not used as the first line of defense against PSD.

TCAs achieve their effects of reducing symptoms of depression by working on the pathways of several neurotransmitters, including serotonin and norepinephrine. They block the reuptake of the chemical messengers in the presynaptic terminals, leading to their increased concentration in the synaptic cleft. The three-ring structure of TCAs, attached to secondary (inhibits reabsorption of norepinephrine) and tertiary (inhibits reabsorption of serotonin) amines, makes it possible for the antidepressants to cause a blockage of the neuronal pathways. The high volume of serotonin and norepinephrine enables TCAs to efficiently perform their anti-depressive effect by enhancing mood [76,102]. They are primarily administered in the form of oral tablets, capsules, or solutions, with the oral route being the primary administration method. Patients can take low doses to start and gradually increase them as necessary.

However, despite their efficacy, TCAs also have a high risk of adverse effects due to their varying degrees of receptor affinities. For instance, they block the effects of histamine, leading to drowsiness, confusion, increased appetite, and blurred vision [80]. They also cause blockage of cholinergic receptors, thus making individuals experience problems such as urinary retention, xerostomia, and tachycardia [102]. Additionally, it causes complications such as ventricular fibrillation and sudden cardiac death among PSD individuals with preexisting ischemic heart disease. Antidepressants can contribute to stroke recurrence and increase suicidal ideation in persons aged 24 and below [102]. Doctors and physicians should therefore be extra cautious when prescribing TCAs to minimize the adverse impacts of the drugs.

TCAs have been widely studied for their efficacy. Deng et al. [103] performed a meta-analysis of 23 RCTs of nine common interventions used in managing PSD. The results showed that TCAs are among the best approaches to managing post-stroke depression, alongside SSRIs and SNRIs. Tan et al. [104] investigated the efficacy of citalopram in PSD treatment compared to TCAs and other drugs. The results showed that TCAs had a higher efficacy index than citalopram, as per the scores on the Hamilton Depression Scale, with the conclusion that TCAs were more efficient than citalopram in treating PSD. In another meta-analysis, Arroll et al. [105] investigated the efficacy and tolerance level of TCAs and SSRIs in treating depression compared to a placebo. When compared with a placebo, efficacy estimates showed that patients using TCAs had a relative improvement risk. The researchers concluded that it was more effective to prescribe TCAs in primary care than a placebo.

**Table 1:** The results of individual RCTs and meta-analyses for PSD treatment.

Author	Number of Studies	Study Design	Study Approach	Results	Conclusion
Sun et al. 2017 [77]	12 suitable trials, with data from 707 participants	Multiple treatments meta-analysis of RCTs	SSRIs	The results established that the acceptability scores for doxepin, citalopram, and fluoxetine were higher than paroxetine.	Paroxetine was the preferred drug at the onset of acute treatment for PSD, and fluoxetine was the least ideal choice.
Li and Zhang 2020 [78]	Data from 51 RCTs	Multiple treatments meta-analysis of RCTs	SSRIs, TCAs, mirtazapine	The results showed that escitalopram was the most favorable, while amitriptyline scored the lowest at two weeks. Citalopram was more effective than the other SSRIs at four weeks, while mirtazapine had the highest response rate among patients after eight weeks.	Escitalopram was associated with a quicker relief of depression, but mirtazapine was probably the best option for an 8-week treatment duration.
Zhou et al. 2020 [73]	Data of 10 RCTs with a total of 5370 patients	Multiple treatments meta-analysis of RCTs	SSRIs	The results showed that SSRI therapy was most effective in preventing PSD when used in the initial stages of the condition.	Early SSRIs therapy was effective in preventing post-stroke depression. However, SSRIs did not improve patients’ post-stroke functional independence. SSRIs were relatively safe.
Zhang et al. 2013 [85]	95 patients	RCT	SNRI: Duloxetine	Duloxetine helps stroke patients prevent minor and major depression and also helps them rehabilitate rapidly from a stroke.	Prophylactic use also reduced the incidence of PSD and improved individuals’ quality of life and cognitive function.
Cravello et al. 2009 [86]	50 patients	RCT	SNRI: Venlafaxine	The results showed that the patients had more significant improvement in alexithymia than those treated with fluoxetine.	Antidepressants **such as** SNRIs were effective in treating depression and improving emotional awareness in PSD patients.
Tsai et al. 2011 [87]	92 patients	RCT	SNRI: Milnacipran	The results showed that milnacipran had a significant advantage in preventing PSD without substantial adverse effects.	Preventive use antidepressants **such as** SNRI (milnacipran) reduced incidences of PSD.
Patel et al. 2016 [92]	Data from 51 RCTs	Multiple treatments meta-analysis of RCTs	Bupropion	The results showed that bupropion was effective and highly tolerated. Patients using it had low rates of side effects.	The researchers concluded that bupropion since it had equivalent effectiveness to other antidepressants.
Liu et al. 2015 [93]	Data from 9 RCTs and 1,106 patients	Multiple treatments meta-analysis of RCTs	SNRIs: Fluoxetine Bupropion	The results showed no difference in effectiveness between bupropion and other antidepressants **such as** fluoxetine.	Bupropion hydrochloride sustained-release tablets have the same effectiveness and side effects as fluoxetine tablets in the treatment of depression.
Qin et al. 2018 [101]	Data from 14 RCTs included 949 patients	Multiple treatments meta-analysis of RCTs	MAOs SSRIs TCAs	The results showed insufficient evidence that MAOIs were more effective and tolerant than placebo and other antidepressants **such as** SSRIs.	MAOIs are less effective than SSRIs.
Deng et al. 2017 [103]	Data from 23 RCTs included 1542 patients	Multiple treatments meta-analysis of RCTs	NDRIs SSSIs TCAs	The results showed that TCAs are among the best approaches to managing post-stroke depression, just **such as** SSRIs and SNRIs.	NDRIs, SSRIs, and TCAs are associated with a considerably higher depression score reduction compared with the control groups.
Tan et al. 2015 [104]	Data from 48 RCTs included 3294 patients	Multiple treatments meta-analysis of RCTs	SSRIs TCAs	The results showed that TCAs had a higher efficacy index than citalopram as per the scores on the Hamilton Depression Scale.	They concluded that TCAs were more efficient than citalopram in treating PSD.
Arroll et al. 2005 [105]	Data from 12 RCTs included 2753 patients	Multiple treatments meta-analysis of RCTs	SSRIs TCAs	The results showed that when compared with a placebo, efficacy estimates showed that patients using TCAs had a relative improvement risk.	The researchers concluded that it was more effective to prescribe TCAs in primary care than a placebo.

### 5.2. Glutamate-Based Antidepressants

Glutamate-based antidepressants are drugs designed to correct glutamatergic dysfunction, which forms part of the pathophysiology of depression. These types of drugs are used when PSD patients fail to respond positively to antidepressant medications, such as SSRIs and SNRIs, that act primarily on the monoaminergic system. Studies show that depression drugs based on the monoamine theory are only effective in approximately two-thirds of patients [106]. It is possible that a combination of glutamate-based antidepressants and monoamines provides the most ideal treatment option [107].

Given that glutamate is the primary excitatory chemical messenger in the CNS, the receptor subtypes of the glutamatergic system have highly been involved and applied in developing more effective treatments for mood disorders. The excitation occurs because agonist binding occurs at the postsynaptic receptors [106]. The receptors are divided into three types: ionotropic, consisting of N-methyl-D-aspartate (NMDA), α-amino-3-hydroxy-5-methyl-4-isoxazole propionic acid (AMPA), and kainite receptors; and metabotropic, consisting of group I, II, and III metabotropic glutamate receptors (mGluRs) [106].

#### 5.2.1. NMDA Blockers

NMDA blockers, known as NMDA antagonists, are a category of medications that treat diseases such as PSD, which can cause adverse effects such as poor functional ability and negative quality of life. NMDA antagonists perform their role by binding to NMDA receptors while preventing the glutamate from binding to the site [108]. As a result, they prevent the release of calcium into the nerve cells, which is essential in memory. Excess calcium, released after glutamate attaches to the NMDA receptor, can cause severe damage to the vital nerve cells [108]. The most commercially available NMDA blockers are ketamine, memantine, magnesium, and D-cycloserine.

##### Ketamine

Ketamine is a non-selective NMDAR channel blocker that has been reported to inhibit catecholamine reuptake. The drug interacts with several CNS targets such as opioid, sigma, and muscarinic receptors to produce rapid and robust antidepressant effects in PSD [109]. It antagonizes NMDA receptors on GABAergic interneurons, thereby disinhibiting afferents to the primary glutamatergic neurons. It, therefore, induces an acute decline in glutamate release in the brain, thus minimizing the overexcitement of nerve cells. Increased excitation of the cells heightens the risk of brain cell damage or death. Therefore, ketamine stabilizes the glutamatergic system, whose dysfunction causes mood disorders, including PSD [110].

Ketamine is usually used as a dissociative anesthetic. There are several ways in which the drug can be administered. Ketamine can be sublingual, oral, intramuscular, saline, transmucosal, intranasal, and intravenous. Abdoulaye et al. [111] established that the administration of ketamine aids in faster and more positive impacts on depressive symptoms. The injection of ketamine in individuals suffering from PSD could increase the concentration of atrial natriuretic peptide synthesized in atrial myocytes, thereby exerting anti-depression effects [112]. PSD patients usually have lower plasma atrial natriuretic pepide levels and an attenuated N-terminal prompt response to chronic stressors than healthy individuals.

Patients react differently to ketamine medication. There are some who will exhibit a more significant reduction in depressive symptoms within 24 h after a single infusion of ketamine [113]. Others with treatment-resistant depression will not see immediate changes after taking the drug. In such a case, repeated ketamine administration has to be provided for the patients to experience cumulative and sustained antidepressant effects [113]. Thus, ketamine’s mechanism of regulating the volume of glutamate in the brain makes it possible for it to perform mood modulation, treating the PSD in the process.

As a non-competitive antagonist of NMDA, the use of ketamine has proved to be effective and contributes significantly to clinical improvement in depressive symptoms. Despite its efficacy, however, ketamine can also cause mild side effects in patients. Phillips et al. [113] report numbness, tingling, and dizziness are some of the reactions caused by the medication. Moreover, it has been associated with transient cognitive deficits, increased blood pressure and libido, and perceptual disorders among patients with treatment-resistant depression [114].

##### Memantine

The drug, which is a non-competitive NMDA receptor antagonist with moderate affinity, is approved for both moderate and severe forms of depression. A research study by Lopez-Valdes et al. [115] showed that memantine improves stroke outcomes in a non-neuroprotective manner. The drug increases brain-derived neurotropic signals and improves vascularization, which helps recover sensory-motor, and cortical function impaired by the disorder [115]. Memantine is highly tolerated by many patients and has the capability of improving depressive symptoms and apathy scores [116]. It works by blocking glutamate in the brain, which is generally linked to PSD symptoms. Therefore, memantine positively influences mood, improving cognition and the general quality of life of patients through its action [117].

There is a need for PSD patients to follow special precautions before taking memantine to minimize the side effects. For instance, they should disclose to their doctors whether they are under any other medications, allergic, pregnant, or suffering from a urinary tract infection [116]. It is also vital for individuals to disclose whether they are having surgery, including dental surgery. Some of the mild side effects that this medication causes include constipation, nausea, weight gain, diarrhea, confusion, and sleeplessness [116]. In extreme cases, shortness of breath and hallucinations may result in severe impacts.

##### Magnesium

Low levels of magnesium (Mg) in the body have been linked to the development of depression. Therefore, having a higher concentration of Mg in the body can help improve depressive symptoms. Research studies show that magnesium ions produce cerebral arteriolar vasodilation and increase cerebral blood flow, thereby inhibiting the presynaptic release of excitatory chemical messengers [118]. They also cause the suppression of cortical-spreading depression and acts as a neuroprotective in preclinical models of excitotoxic injury. Consequently, in the brain, Mg blocks the NMDA glutamate receptors, regulating their role in memory formation and learning [118]. When in excess, glutamate neurotransmitters endanger the brain’s health since it causes the overexcitation of cells and eventually leads to their death. As a result, it subjects individuals to the risks of a myriad of medical conditions, such as post-stroke depression, anxiety, and seizures [118].

Since Mg plays a crucial role in regulating the level of glutamate neurotransmitters in the brain, its deficiency predisposes PSD patients to experience increased severity of depressive symptoms [119]. Mg differs from other psychotherapy medications for PSD in that it is a dietary intervention. Patients can obtain it by eating magnesium-rich foods (legumes, milk, nuts, spinach, potatoes, bananas, and whole grains) or taking Mg supplements [120]. A majority of PSD patients have low volumes of magnesium since the disorder induces excessive stress that causes excessive depletion of the essential minerals [119]. Stressors cause the Mg ions to be released into the body before being excreted by the kidneys afterward. The recommended dosage amount depends on patient characteristics, such as age, sex, and pregnancy. However, adult men should take a daily dose of 400 mg and 420 mg, while that of women is 310 mg and 320 mg.

It is infrequent for magnesium to reach toxic levels and hence very safe compared to other NMDA blockers. However, patients can suffer mild side effects, including muscle weakness, lethargy, nausea, diarrhea, and a fall in blood pressure [121]. The risks may be heightened in patients with inherent chronic medical conditions such as impaired kidney function [121]. It is thus crucial for the affected individuals to check with their physicians before taking any supplements.

##### D-cycloserine (DCS)

DCS are antibiotics that are partial agonists at the glycine site of NMDA receptors. DCS has significant antidepressant effects and is well tolerated by patients because they do not produce psychotomimetic effects [106]. DCS works by accelerating the treatment response early in PSD treatment. Their function mode involves blocking NMDA receptors and stimulating the part of the brain responsible for mood and emotional responses [122]. The effect of DCS on glutamatergic activity is limited compared to that of NMDA blocker drugs [123]. In addition to their direct oral administration to PSD patients, DCS can also be used as an extension of exposure-based psychotherapy to reduce the adverse effects of post-stroke depression and associated symptoms.

These drugs are more effective as depression augmentation treatment and are only efficacious when given a limited number of times [123]. It is primarily administered orally at a dosage of between 250 and 500 mg twice daily. Even though it has minimal side effects, high doses can lead to dizziness, hyperexcitability, anxiety, and memory loss. On rare occasions, patients can also experience gastrointestinal problems, fever, cardiovascular troubles, and allergies [123]. However, some of the severe impacts can be mitigated if interactions of DCS with other drugs are considered. For instance, antidepressants such as olanzapine seem to offset the advantageous effects of DCS.

#### 5.2.2. AMPA Antagonists

These neuroprotective drugs belong to the class of excitatory amino acid receptor antagonists. They are anticonvulsants and have effectively treated several medical disorders, including PSD. AMPA is non-competitive and achieves its functions by modulating receptor activity. Anti-excitotoxic drugs counteract excitotoxic mechanisms that cause neurodegenerative disorders such as post-stroke depression. Medicines in this category treat depressive symptoms in PSD patients by either reducing glutamate synaptic levels or blocking the effects of excessive glutamate release on postsynaptic efforts. Riluzole is a common and FDA-approved anti-excitotoxic drug that has proved quite efficacious in patients with treatment-resistant PSD [124]. Anti-excitotoxic drugs intervene in the mechanistic steps that lead to excitotoxicity mediated by glutamate, thereby preventing neuronal death [47]. We have established that the glutamate system has a vital role in the pathophysiology of PSD.

Anti-excitotoxic drugs such as riluzole can effectively perform their role in treating mood disorders such as PSD because of their anticonvulsant and glutamatergic modulating properties. The inhibition of tyrosine phosphorylation, which occurs in response to brain ischemia, enhances the neuroprotective mechanism of riluzole against any form of excitotoxic dysfunction [124]. Different modes of action explain the antidepressant and mood-enhancing effects of riluzole. It is also believed that it transports AMPA; suppresses the HPA axis; and facilitates clearance of glutamate from synaptic space, all of which contribute to the regulation of mood and emotions. Moreover, it can also induce mechanisms linked to G-protein signaling to inhibit glutamate release from presynaptic terminals. The symptoms of PSD patients will improve after six to twelve weeks after medication administration begins. However, treatment can cause several mild side effects, including fatigue, nausea, and weight loss.

#### 5.2.3. Metabotropic Glutamate Receptors

Metabotropic glutamate receptors, commonly known as mGluRs, are G-protein-coupled receptors of the family C that regulate the excitability of neurons and synaptic transmission throughout the central nervous system. It is known that they may bind glutamate inside the extracellular’s larger domain [125]. However, they may also convey signals to intracellular signaling partners through receptor proteins. There are eight unique types of these receptors. They are classified into Groups I, II, and III based on their physiological activity and the shape of their receptors [126,127]. All of these receptors exhibit indirect metabotropic activity. Despite this, there is evidence from behavioural and biochemical studies revealing a solid relationship between glutamate and depression, with a significant and critical connection between glutamate and the treatment of the condition and its etiology [128]. As a result, the regulation of glutamatergic neurotransmission by mGluRs has a significant association with depression. Finding small molecule modulators with the necessary antidepressant characteristics is crucial. Therefore, the receptors provide the opportunity for tailored and comprehensive therapies [128]. Nevertheless, anomalies could have the opposite effect on the situation.

##### Drug Research on Group I mGluR in Depression

mGluR_1_ and mGluR_5_ are Group I mGlURs. The receptors interact considerably with K+ and Na+ channels, and their activities are thought to be excitatory, increasing conductance, hence increasing presynaptic cell glutamate release and IPSPs [129]. Group I mGluRs are mostly linked to the peripheral density of various excitatory synapses. Similarly, it is essential to note that the receptors are primarily activated by DHPG, a critical factor in the research of these two receptors since it permits their separation and identification. Activation often occurs in response to solid activity. It induces the long-term plasticity of synaptic transmission in the cerebellum, neocortex, hippocampus, striatum, and midbrain [130]. In addition, there is substantial evidence of an underlying dependency between the two groups, one of whose receptors are thought to have physiological importance, especially in their co-expression of functioning in a heterologous system.

In light of this, there is overwhelming evidence demonstrating the significance of group I mGluRs compared to drugs acting on them in terms of their ability to cure depression. Negative allosteric modulators (NAMs) of these receptors, including GRN-529, basimglurant, MTEP, and MPEP, have strong antidepressant effects when administered to rats, according to studies [131,132]. Compared to conventional antidepressants, the NAMs of the receptors demonstrate quicker and more effective action while reducing depression’s comorbidities, expediting the treatment of this mental condition. Similarly, research indicates that when the receptors are deleted, an antidepressant-like phenotype emerges, proving that mGluR_1_ and mGluR_5_ exhibit antidepressant effects [133]. DSR-98776 produces quicker and more effective antidepressant effects, as well [134]. MTEP demonstrates that inverse agonist action has the potential to cause significant side consequences, such as psychosis, despite the favorable findings of several studies on pharmacological research involving group I receptors [135]. Partial NAMs, on the other hand, have not shown these benefits, indicating that they are better suited to function as antidepressants.

Nevertheless, postmortem brain and positron emission tomography (PET) have been used severally. Although the receptors in these studies did not demonstrate any substantial changes in depressive patients, considerable receptor depletion was seen in various brain regions, resulting in a compensatory change [136]. In addition, the role of ketamine concerning the two receptors has been evaluated, with data indicating that ketamine considerably lowers the availability of the receptors [137]. This component is related to antidepressant response, leading to the conclusion that the discovered receptors are primarily responsible for ketamine’s antidepressant properties.

Nonetheless, substantial clinical data suggests basimglurant’s significance as an adjuvant therapy for SNRI treatment. Based on the Montgomery-Asberg Depression Rating Scale (MADRS), the NAM is safe [138]. In contrast, the same research found that the NAM resulted in exploratory and secondary endpoint improvement [138]. However, AZD206 did not have any substantial effects on the MADRS modifications. This indicates that adding duloxetine significantly diminishes the antidepressant benefits of this particular NAM [139]. These studies have shown that group I mGluRs have tremendous promise as a treatment for depression and need further investigation.

##### Drug Research on Group II mGluR in Depression

mGluRs2 and mGluRs3 are receptors belonging to group II. These receptors are abundantly expressed and negatively coupled to adenylyl cyclase activity in the limbic and cortical regions. In this context, group II receptors harm the regulation of glutamate transmission in crucial brain areas, especially those associated with emotion and cognition [140]. There is also strong evidence that animal and human emotions fluctuate in the concerned brain region. In addition, animals lacking mGluRs2 exhibit enhanced reward activity and antidepressant-like phenotypes; hence, the two antagonists have been intensively studied for antidepressant-like effects [140].

Decades of the study indicate that NAMs and antagonists of mGlURs2 and mGlURs3 exhibit prolonged and rapid antidepressant-like responses [141]. In animal models, the antagonists LY341495, MGS0039, and LY3020371 have shown effects comparable to those of ketamine, with LY341495 and MGS0039 demonstrating substantial effects during the first 24 h of administration [142,143]. Moreover, the results continued up to a week after a single dosage, indicating that, in addition to being rapid, the antagonists exhibited persistent effects similar to ketamine. Moreover, these antagonists have an advantage over ketamine since there is no evidence associating their use to misuse potential, cognitive impairment, or increased locomotor activity [143]. Similar to RO4491533, acute tests such as TST and FST have shown that RO4491533 can produce antidepressant-like effects [132]. In addition, data shows that activating mGlURs2 may have neurotoxic consequences, but similar activities for the other receptor may generate precognitive benefits, rendering it neuroprotective [132]. Per this, preliminary research on the antagonists of group II mGlURs revealed that antidepressant-like effects were lost in patients devoid of mGlURs2 but retained in those with mGlURs3 [132]. This indicates that the former plays a vital role in the antidepressant-like actions of these receptor antagonists.

In addition, there are ties between L-acetylcarnitine (LAC) and mGlURs2. The agent’s antidepressant-like properties originate from the receptor’s epigenetic modulation. The research revealed an immediate and long-lasting impact on Flinders Sensitive Line mice and rats subjected to unanticipated chronic stress, serving as a model for environmental and genetic depression [144]. In all studied animals, LAC increased the amounts of acetylated H3K27 and NF-kB-p65, therefore boosting the transcription of the gene encoding the mGlURs2 receptor in the prefrontal cortex and the hippocampus [144]. In addition to increasing the desire for sucrose, LAC lowered immobility time without inducing tolerance. Two weeks after the drug’s removal, antidepressant effects were still evident. However, the antagonist LY341495 was observed to inhibit LAC action partly [140]. Despite this, the LAC research presents a novel approach to the epigenetic concept of depression and supports the development of effective and efficient antidepressants [144].

Collectively, these group II data point to the development of more effective and rapidly acting antidepressants for the treatment of depression. In this sense, it can be said with certainty that the receptors in question will provide innovative therapies for depression symptoms. However, further study is necessary for a complete knowledge of the action mechanisms of NAMs and antagonists.

##### Drug Research on Group III mGluR in Depression

The four subtypes of Group III mGluR are mGluR_4_, mGluR_6_, mGluR_7_, and mGluR_8_ [145]. It is known that these receptors are primarily presynaptic, with specific presynaptic terminals down-regulating neurotransmitter release indirectly or directly [125]. In addition, they function as modulatory transmission receptors of gustatory inputs in the brain stem, influencing psychostimulants’ receptive behavior in both the ventral and dorsal striatum [146]. Similar to group II receptors, the coupling of these receptors results in the inhibition of adenylyl cyclase and a considerable decrease in cAMP production [147].

Due to the restricted availability of the specific ligands associated with each of the four receptors, the location of these receptors in depression and their antidepressant potential have not been thoroughly investigated and analyzed [148]. Nevertheless, research has shown that the receptors play a crucial role in GABAergic transmission and glutamatergic modulation in the CNS [149]. These properties make them excellent candidates for consideration as antidepressant and antianxiety medications. Remarkably, mGluR_4_ and mGluR_7_ receptors have been suggested as promising depression therapeutic targets [140].

Primarily, LSP4-2022 has shown considerable potential for inducing pro-depressive effects [149]. ADX88178, a selective and brain-penetrating mGluR_4_ antagonist, has demonstrated strong antidepressant effects [150]. In addition, in the TST and FST tests conducted in this instance, LuAF21934 and LSP1-2111 exhibited anxiolytic effects but no antidepressant effects [151]. Due to the unfavorable influence on the base locomotor activity, caution must take precedence. However, preclinical investigations employing Grm7 in particular research indicated that immobility was decreased compared to TST and FST littermate controls [152]. In addition, the study demonstrated that Grm7 deficiency alleviated chronic stress as well as immunological and physiological stress, underscoring the significance of mGluR_7_ in chronic stress exposure and regulation [152]. As such, Grm7′s deletion alludes to mGluR_7_’s inhibition, making it especially important as a stress treatment [153].

Furthermore, the mGluR_7_ agonist AMN082 has shown tremendous potential for inducing behavioral changes and responses [154]. MMPIP, a selective antagonist of a different receptor, was shown to inhibit the antidepressant-like effects of the agonist significantly [155]. In addition, new research indicates that mGluR_7_ produced in glial cells has neuroprotective effects, demonstrating its antidepressant potential [156,157]. Given the lack of the specific antagonist in Grm7, it suggests that mGluR_7_ entirely regulates the activities and antidepressant-like properties of AMN08230. As a result, the involvement of this receptor is supported by its antidepressant-like effects, which contribute to its effectiveness. To establish definitive claims about the localization and function of mGluR_7_ in the CNS [156], further study and more thorough research methods are required.

In addition, data collected from the gene Grm8, which encodes mGluR_8_, indicates that the receptor plays a crucial role in depression-related pathways [158]. Because the effects of the stressors on the gene were not cumulative, it seems that the receptor in question has regulatory potential in a stressful environment [159]. Despite this, the initial study demonstrates the potential of ligands as therapies for stress-related illnesses such as depression. These ligands are group III mGluRs, which have made substantial progress during the last several years. Significant depressive and anxiolytic-like effects of receptors in this group need more research to clarify their activation, inhibition, and relationship to ketamine.

### 5.3. Blood Glutamate Scavengers

The drugs in this class minimize blood glutamate concentrations, hence preventing dysfunctions that might cause brain injury from occurring. Called blood glutamate scavengers, they increase the pressure of the blood to the brain, influencing the blood glutamate efflux, making it possible to lower the amount of glutamate [160]. When glutamate levels are elevated to an excessively abnormal level, they cause neurogenerative effects that can adversely alter mood and emotions [160]. When the drugs are administered, they act as glutamate co-substrates and convert the excess glutamate into its inactive form 2-ketoglutarate [161]. Hence, close attention is necessary to ensure that the brain glutamate does not rise to a toxic level. The most commonly used drugs in this category that have been approved for the treatment of PSD include pyruvate and oxaloacetate.

#### 5.3.1. Pyruvate

Failure to maintain a balance in glutamate levels can cause deleterious and irreversible complications and neuropsychiatric consequences as it can cause brain injury; PSD is primarily linked to the rise of extracellular glutamate concentrations. Excess glutamate release can cause cell swelling, apoptosis, and neuronal death [161]. Cell damage and cellular dysfunctions may also cause a drop in the levels of glucose in the brain. Thus, a mismatch of cerebral metabolism is highly likely to occur since glucose acts as the primary energy source for brain cells. As a result, the function of neurotransmitters will be impaired, making it challenging to receive and interpret signals needed to comprehend when to sleep, reduce pain, or experience pleasure.

Early administration of pyruvate supplements can help improve cerebral metabolism and neurological outcomes. Hence it is vital for glutamate homeostasis to be maintained for the neurological outcomes of patients to be improved. Studies have established that such danger can be mitigated by using pyruvate as a blood glutamate scavenger [161]. It works effectively by providing neuroprotection to the brain cells. Once the drug is administered, it initiates a reduction of blood glutamate and brain glutamate levels and enhances the regulation of mood and emotions.

PSD patients often have high glutamate concentration in the brain, which is likely the cause of depressive symptoms such as decreased energy, loss of interest, self-blame, and low self-evaluation. Pyruvate has been shown to lower glutamate levels and improve neurological recovery twenty-four hours after middle cerebral artery occlusion [161]. The drug works by eliminating excess toxic glutamate from the brain into the blood. As a result, it manages to mitigate secondary brain injury due to glutamate neurotoxicity. Patients can attain faster and more remarkable improvement of PSD symptoms when pyruvate is administered through intravenous injection.

Pyruvate does not antagonize glutamate receptors but rather eliminates their excess concentration to minimize their excitotoxicity [161]. The mechanism is based on an interaction between pyruvate and glutamate in the blood, which leads to an increasing concentration of alanine and alpha-ketoglutarate. Decreased blood glutamate concentration leads to the creation of a concentration gradient of glutamate between the brain and blood that leads to glutamate efflux, thus inducing the transport of excessive amounts of glutamate from the brain’s extracellular fluid to the blood. Its mode of action is efficient and does not lead to neurological deficits because it does not impact glutamate-mediated synaptic activity or neural circuits whose functionality relies on glutamate transmission.

Pyruvate supplements reduce glutamate concentration by catalyzing the enzymatic process as an acute stress response and redistributing the glutamate into tissue. It is given in single or multiple treatments with sodium pyruvate (SP) or ethyl pyruvate (EP). Both EP and SP treatments produce a rapid response in reducing the number of dying neurons in the cortex and hippocampus within twenty-four hours after administration. By stabilizing the level of glutamate, SP and EP drugs reduce the dying neurons in the brain, thus mitigating the effects of PSD in the process [162]. If administered early, pyruvate has higher efficacy and can reduce glutamate levels by up to fifty percent, resulting in motor improvement and functional outcomes in PSD patients [163]. Therefore, the neuroprotective features of the pyruvate treatment make it possible to attenuate the effects of depression and its associated symptoms in PSD patients.

#### 5.3.2. Oxaloacetate

As a blood glutamate scavenger, oxaloacetate performs a neuroprotective role, enabling it to reduce both blood and brain glutamate levels. Research conducted by Kaplan-Arabica et al. [164] shows that intravenous oxaloacetate administration among stroke patients increases the scavenging activity of the drug. As a result, it significantly helps reduce the concentration of glutamate, thus potentially alleviating the severe symptoms of depression. Stroke patients also exhibit favorable neurological outcomes after being injected with the drug.

It functions by degrading the excess glutamate in the blood to 2-ketoglutarate (its inactive metabolite) through the action of glutamate-oxaloacetate (GOT) coenzymes [9]. In turn, oxaloacetate treatment minimizes secondary brain damage and neuropsychiatric consequences such as post-stroke depression caused by an unprecedented rise in glutamate. Preliminary evidence from clinical trials suggests that using blood glutamate scavengers such as oxaloacetate has no side effects on PSD patients.

### 5.4. Microbiota Treatment in PSD

The human gut and the brain are in constant connection since they use the same hormones and neurons to communicate. The existing association makes it possible for the gut to have a hugely influential role in controlling and preserving emotional and mental balance. The gut is composed of numerous coexisting microorganisms which participate in its functionality. They help in the development and maturity of the brain systems to facilitate stress response and fighting. Most psychological disorders such as depression, stress, schizophrenia, and anxiety are caused by a variation of the microorganisms in the gut system [165]. To treat this, the microbiome is regulated either upwardly or downwardly through the use of prebiotics, probiotics, and antibiotics, depending on the nature of the variation. Brain-gut axis is associated with the decline of microorganisms and thus requires microbiota treatment that involves inducing microorganisms by using probiotics and prebiotics.

### 5.5. Anti-Inflammatory Treatments in PSD

Strokes are known to cause brain inflammation among its patients. This brain inflammation, even though yet to be proven, could be the primary cause of depression after a stroke. In the early periods after suffering from stroke, the brain produces the pro-inflammatory cytokines, which mature after 3–4 weeks following their release. The cytokines are responsible for the activation of the hypothalamus-pituitary-adrenal axis, which in turn increases the level of adrenocorticotropic and cortisol hormones. Additionally, the activation causes the conversation of tryptophan into kynurenine at the expense of serotonin. Due to the serotonin production in the brain following the suppression by tryptophan, the serotonin synthesis is equally lowered, inducing depressive symptoms. The pathway of this depression thus makes it different from the one that occurs later.

The depression that comes immediately after a stroke is pathogenic. The pathogenesis causing the induction of depression has inflammatory components, hinting it could be suppressed or mitigated by using anti-inflammatory drugs such as acetylsalicylic acid. Non-steroid anti-inflammatory medications with the ability to suppress inflammation, therefore, qualify as an alternative treatment for depression after stroke [166]. Certain anti-inflammatory drugs such as acetylsalicylic drugs and statins have proven to mitigate and lower the risk of depression after stroke by suppressing inflammation in the brain. It is possible that non-steroid anti-inflammatory (NSAIDs) medications with anti-inflammatory properties can also be administered to aid in treating depression after a stroke. It is, however, essential to note that anti-inflammatory drugs such as NSAIDs are only functional in some types of stroke. While most stroke victims are likely to suffer depression following their recovery from the event, not all depressive symptoms are caused by brain inflammation; thus, not all can be treated by NSAIDs.

PSD occurs in two phases: immediate depression, which happens immediately after recovery from stroke; and later depression, which occurs after recovery from stroke. For victims that suffer depression immediately after recovery from stroke, depression can be suppressed by using NSAID drugs as the depression is usually caused by brain inflammation. Thus, the anti-inflammatory property of the NSAIDs suppress brain inflammation and thereby mitigate depression [167]. NSAIDs such as ibuprofen, naproxen, and diclofenac, among others, may be used to treat PSD. Due to their availability and affordability, many individuals are now considering NSAIDs as alternative antidepressants. Common NSAIDs such as diclofenac, celecoxib, and aspirin, among others, have recently been considered adequate alternative antidepressants.

### 5.6. Other Drugs

Mirtazapine and agomelatine are some of the alternative drugs used in the treatment of PSD. They are taken as adjuvant medicine to improve PSD management. Most of these medications are usually prescribed to help reduce drug dependence caused by antidepressants [84]. However, only a few studies have focused on the efficacy of these treatment methods and their potential antidepressant effects.

#### 5.6.1. Mirtazapine

Mirtazapine is considered a first-line treatment of post-stroke disorder along with medications such as SSRIs and SNRIs. It is a selective, peripheral-alpha1-adrenergic and muscarinic receptors, presynaptic alpaha2-adrenergic, 5-HT3 serotonin, and H1 histamine antagonist [168]. It has a dual mode of action, as both a noradrenergic and specific serotonergic depressant [169]. Its functional mechanism increases the release of norepinephrine and serotonin, which mobilize the brain to take control of its functions. A high concentration of norepinephrine improves the energy and attentiveness of depressed patients, while serotonin regulates mood. By preventing the depletion of these two essential neurotransmitters and maintaining their balance, mirtazapine is thus able to prevent a return of depressive symptoms in PSD patients. As a prophylactic treatment, clinical studies show that mirtazapine has demonstrated great success as an acute treatment for PSD, with fewer anticholinergic and cardiovascular side effects [170]. It mainly exists in the form of an oral tablet taken by mouth with or without food and is usually taken once daily at bedtime. Despite its advantages, mirtazapine has side effects such as increased appetite, weight gain, headache, nausea or vomiting, diarrhea, and constipation (Table 2).

Individuals are expected to take the same time each day to benefit from it most. The dosage (30–45 mg/d) varies in each patient based on the severity of their conditions and response to treatment, with the improvement of symptoms being noticed between 1 and 4 weeks. It can also be combined with other medications, especially in patients exhibiting partial responses to antidepressant monotherapy after at least six weeks. The augmentation is carried out based on the current medicines of patients and comorbid conditions of antidepressants.

#### 5.6.2. Agomelatine (AGM)

The drug is sold under the brand names Valdoxan and Thymanax. They are highly effective antidepressants and significantly help discontinue the symptoms of post-stress depression. AGM is a melatonergic antidepressant whose mechanisms for improving the depressed states of PSD patients involve the resynchronization of perturbed biological rhythms. The synergistic agonist properties of these drugs at the melatonin receptors also largely contribute to their short-term and long-term efficacy in treating PSD [171]. The melatonergic receptors, which include MT1 and MT2 localized in the cerebral cortex, thalamus, and hippocampus, significantly influence circadian rhythms, such as disrupted sleep-wake cycles. AGM thus stimulates the melatonin receptors, making it possible to alleviate the effects of PSD by improving sleep quality and functional mobility. Consequently, the drugs induce the suppression of 5-HT_2C_ neurotransmission, which inhibits the release of noradrenaline and dopamine [171]. As a result, the level of dopamine and noradrenaline in the frontal cortex of PSD patients increases, inducing therapeutic effects on depressive symptoms.

AGM mostly comes in the form of tablets and is administered orally. Patients should take it consistently at bedtime for maximum benefits to be realized. Dosage may only be increased after two weeks of treatment when PSD patients fail to show improvement in symptoms [172]. The treatment duration is six months to ensure that individuals are fully treated and free of depressive symptoms. However, treatment can cause several side effects, including anxiety, nausea and vomiting, stomach pain, insomnia, dizziness, fatigue, and an increase in weight. Doctors must perform liver function tests to check for hepatic impairment in all their patients before commencing the treatment since the medication can cause mortality in people with liver problems.

#### 5.6.3. Psychostimulants

The use of psychostimulants such as methylphenidate (Ritalin) as an alternative treatment drug for mood disorders, including PSD, has gained traction over the years. The literature suggests that Ritalin can help improve PSD symptoms such as apathy and lethargy [173,174]. Ritalin causes therapeutic effects on cognition, motivation, and mood by enhancing the level of chemical transmitters such as dopamine and noradrenaline [175]. It inhibits the reuptake of these beneficial neurotransmitters from the brain, thereby allowing them to stabilize the mood of affected individuals successfully. It is advantageous compared to other antidepressants because it produces rapid responses [176].

Once the treatment is initiated at a recommended dose of 17 mg/d, patients may find improved symptoms within three to ten days based on their severity. Short treatments of methylphenidate help normalize blood oxygenation level-dependent responses in neuronal networks, invoking improved functional performance and mood.

#### 5.6.4. Nootropic Drugs

The drugs in this category are also referred to as cognitive enhancers. They improve memory and mental alertness, boosted energy levels, and reduce sleeplessness among patients suffering from depression [177]. Research evidence shows that nootropics may simultaneously act on various systems, including increasing the number of neurotransmitters such as dopamine in the brain. The drugs are classified into eugeroics, ADH medications, and nootropic supplements. Eugeroics are primarily used in treating disorders such as PSD since they help prevent depressive symptoms in patients by promoting wakefulness and alertness [178]. Modafinil is one of the most commonly used Eugeroic drugs and exists in brand names such as Modavigil, Modafinil, and Nuvigil.

Modafinil is combined with other antidepressants to reduce the severity of PSD depressive symptoms effectively. The drug works by stimulating a number of neurotransmitters, including histamine, norepinephrine, serotonin, dopamine, and oxygen in the brain to bring about its alerting effects. For instance, if it stimulates serotonin production, the ability of the brain to positively regulate mood is enhanced, making PSD patients feel more focused, calmer, and emotionally stable [179]. However, the affected individuals must seek directions from their physicians when taking the drug since it can cause side effects such as headaches, chest pain, nausea, and nervousness.

#### 5.6.5. The Contribution of Cyclooxygenase-2 (COX-2) Inhibitors in Depression and Ischemic Brain Injury

Cyclooxygenases are known to catalyze the first step in prostaglandins formation from arachidonic acid leading to the production of reactive oxygen species in the process. COX-2, the inducible isoform, is expressed within the brain but is often rare or absent in specific organs under normal conditions. Its regulation can always be tied to inflammatory cytokines and increased prostaglandin production in response to CNS’s pathological conditions [180]. Its overexpression is also inked to exacerbation of given neuroinflammatory responses. Usually, COX-2′s action leads to an end product known as prostaglandin E2 (PGE2) which holds a critical role in the retrograde regulation of the glutamate level.

Further still, COX-2 inhibitors are known to affect the CNS serotonergic system directly and indirectly through its immune processes. A study on rats asserted significant serotonin levels in the temporoparietal and frontal cortices post-rofecoxib administration [181]. This led to the development of the hypothesis that COX-2 inhibitors are likely to have specific antidepressant impacts. In another study that involved bulbectomized rats, celecoxib’s chronic administration led to a reduction in the levels of cytokine and showed a significant alteration in the behavior of the subject animals [181]. Another study focused on ASA found that there was accelerated fluoxetine’s antidepressant effect. In human subjects diagnosed with major depression, a comparison was made between reboxetine plus celecoxib. The study established that celecoxib had superior antidepressant impacts on patients with elevated IDO activity levels [182]. Another study compared fluoxetine and celecoxib, and those patients receiving adjunctive celecoxib were reported to have better outcomes [183], with similar results reported in a separate study that looked at sertraline plus celecoxib [184]. There was also evidence of the essence of COX-2 inhibitors in depression treatment in line with NSAID [182]. This concluded that celecoxib had great potential to act as a treatment for this mental disorder, with its safety and benefits quite promising [182]. Another meta-analysis on inflammation-related treatment procedures found that such methods positively affected depression and depressive symptoms treatment [185]. Collectively, these studies assert that COX-2 inhibitors positively influence the treatment of depression, particularly celecoxib. It has been proven that it hastens current treatment without severe side effects [185,186,187]. However, there is still a need to investigate further COX-2′s inhibition effects in the CNS and towards significant components such as glutamatergic neurotransmission and kynurenine metabolism.

Similarly, COX-2 inhibitors provide a valuable therapeutic target regarding ischemic brain injury treatment. These inhibitors can attenuate injury in stroke models while producing a prostanoid synthesis unbalance, which promotes harmful vascular effects [181]. In particular, COX-2 inhibitors reduce cell death and inflammation while enhancing behavioral recovery even when the administration is conducted hours after the injury. The evidence is suggestive that the COX-2 inhibitors have an extended opportunity window of protecting the brain’s vulnerable tissues from secondary damage. Furthermore, studies suggest that COX-2 inhibitors are more prominent than free radicals and prostaglandins reduction [181]. The inhibitors are known to increase epoxyeicosatrienoic and hydroxyeicosatetraenoic acids levels while also shunting the arachidonic acid in the injured brain area [188]. The metabolites from these are prime candidates for neuroprotective eicosanoids. Moreover, there is evidence that COX-2 inhibitors with safer cardiovascular profiles are likely more suitable and stable. These include third-generation inhibitors and lumiracoxib [188]. Therefore, the continued successes of these inhibitors offer great potential in ischemic brain injury treatment with powerful tools to improve functionality and lessen any arising damage.

##### The Link between COX-2 and mGluR7 in Depression and Cognition

The expression of COX-2 in the brain has been substantially linked to inflammation. It is also extensively expressed throughout the glia and neurons of the brain, and it plays a significant role in many of the essential activities of the brain, such as the consolidation of memories or the plasticity of synapses [189]. Since mGluRs play a role in behavioral and plastic changes, there is an intrinsic interaction between them and COX-2. Glutamate levels are significant for mGluR7, which is often presynaptically localized. Glutamate levels determine whether or not mGluR7 is activated, with activation occurring when glutamate levels are increased. To demonstrate the connection between the two, researchers administered NS398 and MTEP to mice in the same experiment. As a result of this, the receptor in issue had diminished to a large degree [190]. The changes obtained within an hour after the experiment suggested that COX-2 inhibition leads to a decrease in mGluR7 protein. The findings after 24 h seemed to be maintained because of the induction caused by the combination of MTEP and NS398 therapy [190]. As a result, a single injection did not generate a reduction in the number of receptors, but repeated injections were responsible for the observed level of receptor reduction. As a result, there is an undeniable link between the down-regulation of mGluR7 and cognitive impairment. Despite this, participants in the research showed impaired learning after receiving frequent injections of NS398 + MTEP [156,189]. These symptoms included impaired spatial learning in conjunction with a lack of antidepressant-like effects detected in the receptor. Nevertheless, the control of synaptic activity may be accomplished by GABA or glutamate.

As a result, the down-regulation of mGluR7 that occurs after treatment with MTEP and NS398 is a glutamate transmission modulator [156]. However, the precise interaction that results from the combination of COX-2 and mGluRs is not as clear regarding the downstream processes and membrane fluidity that are similar to mGluR7 agonists and also occur as a result of COX-2 inhibition and mGluR5 blockage.

## 6. Results of Non-Pharmacological Treatments

### 6.1. Cognitive-Behavioral Therapy

The use of cognitive behavioral therapy (CBT) in the treatment of PSD has increased recently despite the inconsistency in the findings about its effectiveness. While some studies have found that CBT has more efficacy in reducing depressive symptoms, contrasting results have also been evident, hence the need for further research.

Nguyen et al. [191] did a randomized controlled trial with 15 participants to determine the effectiveness of CBT in minimizing post-stroke fatigue. Nine people underwent weekly eight sessions of CBT, while 7 received usual rehabilitation care. The results show that those who receive CBT treatment experience improved PSD symptoms and post-stroke fatigue compared to regular treatments. In another randomized controlled trial by Gao et al. [192], 2113 study subjects were placed into three groups: one received a placebo, the second received citalopram, and the third received CBT. Patients who underwent CBT had a delayed onset of PSD symptoms. Kootker et al. [193] performed a randomized controlled study using 61 participants placed into a group of computerized cognitive training (CCT) or CBT to determine which of the two modalities had more benefits in reducing PSD symptoms. No significant improvement in symptoms was found between CBT and CCT. This study contrasted the previous findings that CBT is more effective than other treatment mechanisms for PSD.

### 6.2. Electroconvulsive Therapy

Electroconvulsive therapy (ECT) is rapidly emerging as a non-pharmaceutical treatment for various mental health illnesses, including for the improvement of depressive symptoms in patients with PSD. The intervention for this modality entails the passage of electric current through the brain, mainly carried out under general anesthesia. 

An RCT by Cai et al. [194] aimed to identify the effectiveness of electroacupuncture (EA) and ECT in reducing depressive symptoms after stroke. Sixty-two study subjects were recruited into two groups for 12 weeks of therapy sessions. Results reveal the efficacy of both ECT and EA in improving PSD symptoms, thus supporting the use of this mechanism in future treatments. A study by Semkovska et al. [195] compared the effectiveness of high-dose bilateral ECT with unilateral ECT in reducing depressive symptoms. The researchers assigned 69 participants to each group, with results showing no significant differences in scores on the Hamilton Depression Scale. Hence, bilateral ECT is not superior to unilateral ECT in lowering depressive symptoms. A meta-analysis by Van Diemen et al. [196] further affirmed that ECT is the best treatment for severe depression in the elderly with a history of strokes. A review of 2193 articles demonstrates that ECT can lower the recurrence of symptoms in patients with depression when effectively delivered. The study concluded that similarly to the previous findings, ECT is an effective non-pharmacological treatment for PSD.

### 6.3. Transcranial Direct Current Stimulation (tDCS)

The tDCS is an emerging non-pharmacological treatment method for depression. It entails stimulating the brain using a non-invasive procedure, where a direct current goes through the regions that regulate moods and emotions. The aim is to modulate neuro-activity, resulting in better mood balance. As a new treatment modality, many studies are still ongoing to determine its effectiveness.

A systematic review of RCTs by Marchina et al. [197] utilized seven articles containing 217 patients to determine the benefits of tDCS on depressive symptoms after stroke. The results showed that after the tDCS sessions, patients had a low score on the depression scale, indicating that the method is effective in PSD. In another randomized control study, Bornheim et al. [198] sampled 50 patients with a history of stroke to determine the efficacy of tDCS on sensory and functional outcomes. After three months to a year, there were clinical improvements in the functional motor outcomes after tDCS, indicating the efficacy of this technique in improving depressive symptoms. Similarly, Valiengo et al. [199] sampled 48 stroke survivors in a randomized controlled trial to examine the beneficial impacts of tDCS on depression after stroke. The results indicate that those who received tDCS scored low on the Hamilton Depression Rating scale, thus affirming previous studies that the method remains beneficial in PSD. It can be hypothesized that tDCS therapy stimulates the parts of the brain responsible for moods, leading to better emotional regulation.

### 6.4. Repetitive Transcranial Magnetic Stimulation (rTMS)

The use of rTMS in treating depression is relatively new. The procedure entails a non-invasive delivery of magnetic pulse to the brain using electromagnetic activity. The goal is to stimulate the part of the brain involved in depression and mood control. As an emerging area, research on its efficacy remains inconclusive.

In a meta-analysis, Shen et al. [200] reviewed 22 RCTs involving 1764 patients to determine the efficacy of rTMS in reducing depressive symptoms in patients with PSD. Many RCTs revealed that rTMS can positively lower depression in patients with a history of stroke, which was reflected in the Hamilton Depression Rating Scale. At the same time, Hardacre et al. [201] analyzed 11 stroke survivors in a randomized controlled trial to investigate the therapeutic efficacy of rTMS in reducing symptoms of depression. The researchers used the Beck Depression Inventory score, with findings showing that those who received rTMS scored lower. It reveals that rTMS can significantly improve depressive symptoms after stroke. A meta-analysis of 6 RCTs by Shao et al. [202] also found that undergoing a one-month rTMS can positively lower the emergence of depressive symptoms after stroke. In these studies, patients who underwent rTMS scored lower on the depression scale than those treated with typical treatment mechanisms.

### 6.5. Vagus Nerve Stimulation (VNS)

The therapeutic treatment of vagus nerve stimulation (VNS) involves the use of a device implanted in patients with severe treatment-resistant depression. The device causes intermittent electrical stimulation of the left cervical vagus nerve. This treatment is approved in the United States and Europe only for long-term treatment of depression in patients who have not responded to four antidepressant treatments. A relatively new modality, VNS continues to be reviewed and critiqued for its efficacy [203].

## 7. Future Research in Drug Therapy of PSD

PSD is one of the common disorders after stroke associated with poor quality of life, mortality, and a higher risk of suboptimal recovery among stroke survivors. However, despite its harmful impacts, uncertainty remains regarding the optimal strategies that should be implemented for prevention and treatment. The current literature on PSD has primarily focused on pharmacological mechanisms for managing the disorder. Most of the proposed composite treatment regimens have failed to provide desirable results since they expose a patient to myriad side effects that further deteriorate their health and increase the risks of other complications. Therefore, there is a need for future research in drug therapy to focus on integrative strategies that combine both pharmacological and non-pharmacological PSD interventions. Non-pharmacological interventions should primarily include educational, mental, psychological, and physical health support offered to patients, caregivers, or families to enhance their health outcomes.

Interventions such as psychological care are concerned with understanding the needs of patients and offering timely support. Working hand-hand with individuals affected by PSD through their recovery journey makes them realize that they are not alone. This initiative will go a long way in reducing the possibility of patients contracting other psychological issues such as anxiety and improving their health outcomes [204]. Such can be achieved by people close to the suffering individuals and caregivers engaging in active roles such as volunteering, support group attendance, or attending joint physical activities. Moreover, listening to patients’ thoughts without being judgmental can go a long way in offering encouragement and improving recovery among persons with mild symptoms. Promoting creative and simulating activities will enable patients to re-learn functional skills. They will also have the opportunity to keep up with their old hobbies and feel more confident and validated in their abilities to complete a simple task with limited external assistance. Acknowledging their achievements, no matter how small, will motivate patients to keep working hard to improve their conditions, thereby adding to significant victories.

Pharmacological interventions are sometimes recommended when a patient has already suffered significant neuronal damage, making it difficult for them to recover quickly. More effort should be directed toward the early detection of individuals at risk to initiate individualized, preventive measures at the initial stages of PSD. Such a move will reduce mortality rates and minimize the medical and sociological burden associated with the conditions. Rehabilitation of the affected individuals takes significant time, and substantial financial resources incurred in prolonged treatment can take a toll on the finances of the family involved. Woranush et al. [205] established that the efficacy of post-stroke depression increases with the duration of preventive treatment. Therefore, improved outcomes will be realized if the medications used provide rapid and robust responses.

Delayed recovery of PSD predominantly occurs because most randomized control trials of the drugs are not well designed to test efficacy and safety. The FDA should take an active part in analyzing the potential side effects of manufactured drugs. Approval should only be allowed for medications with limited risks that are incapable of preventing PSD from recurring. Consequently, the effectiveness of personalized non-pharmacological treatment options and therapies such as acupuncture should be explored to ascertain their efficacy in treating and mitigating the effects of PSD. Clinical therapies for post-stroke depression are currently dominated by SSRIs, SNRIs, and TCA. These drugs can increase the risk of problems such as sexual dysfunction, severe insomnia, and cerebral hemorrhage [206]. There are also potential risk factors for drug-food interactions and drug-drug interactions, which minimizes their efficiency and safety. Therefore, treatment methods such as acupuncture, exercise interventions, and cognitive and behavioral therapy should be combined with drugs to reduce the effects and improve the depressive symptoms of PSD patients.

## 8. Conclusions

This review has provided a detailed analysis of the current knowledge of new drug treatments for PSD. PSD creates both physical and psychological difficulties for stroke survivors. The disorder affects millions of people, adversely affecting their everyday life. Some common problems experienced by most patients include lower quality of life, impaired motor functions, and mortality. PSD has a complex pathogenesis that involves neurotransmitters found in the brain. A wide array of research shows that significant progress has been achieved in managing and treating PSD and its associated symptoms. Here, we demonstrated that pharmacological interventions are the most widely used to curb the harmful effects of PSD. SSRIs, SNRIs, and TCA classes of drugs are the most popular and are used as a first-line treatment due to their efficacy. NMDA blockers and AMPA antagonists are other drugs that have significantly helped improve the outcomes of PSD patients. The drugs are usually prescribed based on the condition of the affected individuals and the part of the brain where the neurotransmitters are affected. Through this review, it is evident that pharmacological treatment methods present many side effects to patients despite their effectiveness in treating PSD. Thus, there is a need for future research to address ways in which non-pharmacological and pharmacological interventions can be combined to reduce the risks of the problems highlighted.

## Figures and Tables

**Table 2:** The table of advantages and disadvantages of various antidepressant treatments.

Antidepressants	Advantages	Disadvantages
SSRIs	GAD, fibromyalgia	Sexual dysfunction, nausea, vomiting, insomnia, serotonin syndrome, HTN
SNRIs	GAD, fibromyalgia	Sexual side effects, insomnia, nausea, vomiting, HTN, serotonin syndrome
NDRIs	Alcohol, smoking cessation, no sexual dysfunction, no weight gain	Anxiety, suicidal ideation, seizures, general side effects
MAOIs	Effectiveness for atypical depression, PTSD	Low sex drive, weight gain, high or low blood pressure, keep off during alcoholism, kidney and heart disease, food restriction
TCA	Proven efficacy, low cost	Urinary retention, xerostomia, tachycardia, VF, SCD
Ketamine	Severe, treatment-resistant depression, increasing libido	Numbness, tingling, dizziness, transient cognitive deficits, and increasing blood pressure
Memantine	Moderate and severe form depression, highly tolerated by many patients, improving cognition and general quality of life	Constipation, nausea, weight gain, diarrhea, confusion, sleeplessness, shortness of breath, and hallucination
D-cycloserine	Well tolerated, do not produce psychotomimetic effects	Dizziness, hyperexcitability, anxiety, memory loss, and gastrointestinal problems
Magnesium	Anxiety, anticonvulsive effect	Muscle weakness, lethargy, nausea, diarrhea, and a fall in blood pressure
Riluzole	Anticonvulsive effect	Fatigue, nausea, and weight loss
Mirtazapine	Can be used as a preventive treatment for PSD	Increased appetite, weight gain, headache, nausea or vomiting, diarrhea, and constipation
Agomelatine	Short-term and long-term efficacy in treating PSD	Hepatic impairment, anxiety, nausea and vomiting, stomach pain, insomnia, dizziness, fatigue, and an increase in weight

## Data Availability

Not applicable.
